# Current Progress and Future Directions of Enzyme Technology in Food Nutrition: A Comprehensive Review of Processing, Nutrition, and Functional Innovation

**DOI:** 10.3390/foods15020402

**Published:** 2026-01-22

**Authors:** Yu-Yang Yao, Yuan Ye, Ke Xiong, Shu-Can Mao, Jia-Wen Jiang, Yi-Qiang Chen, Xiang Li, Han-Bing Liu, Lin-Chang Liu, Bin Cai, Shuang Song

**Affiliations:** 1 Beijing Engineering and Technology Research Center of Food Additives, Beijing Technology and Business University (BTBU), Beijing 100048, China; 2 Beijing Key Laboratory of Flavor Chemistry, Beijing Technology and Business University (BTBU), Beijing 100048, China; 3 Beijing Laboratory for Food Quality and Safety, Beijing Technology and Business University (BTBU), Beijing 100048, China; 4 Beijing Innovation Centre of Food Nutrition and Human, Beijing Technology and Business University (BTBU), Beijing 100048, China

**Keywords:** enzyme technology, food processing, nutritional enhancement, functional foods, bioactive peptides, enzyme immobilization, precision nutrition, sustainable food processing

## Abstract

Enzyme technology, characterized by high efficiency, environmental compatibility, and precise controllability, has become a pivotal biocatalytic approach for quality enhancement and nutritional improvement in modern food industries. This review summarizes recent advances and underlying mechanisms of enzyme applications in food processing optimization, nutritional enhancement, and functional food development. In terms of process optimization, enzymes such as transglutaminase, laccase, and peroxidase enhance protein crosslinking, thereby markedly improving the texture and stability of dairy products, meat products, and plant-based protein systems. Proteases and lipases play essential roles in flavor development, maturation, and modulation of sensory attributes. From a nutritional perspective, enzymatic hydrolysis significantly improves the bioavailability of proteins, minerals, and dietary fibers, while simultaneously degrading antinutritional factors and harmful compounds, including phytic acid, tannins, food allergens, and acrylamide, thus contributing to improved food safety and nutritional balance. With respect to functional innovation, enzyme-directed production of bioactive peptides has demonstrated notable antihypertensive, antioxidant, and immunomodulatory activities. In addition, enzymatic synthesis of functional oligosaccharides and rare sugars, glycosylation-based modification of polyphenols, and enzyme-assisted extraction of plant bioactive compounds provide novel strategies and technological support for the development of functional foods. Owing to their high specificity and eco-friendly nature, enzyme technologies are driving food and nutrition sciences toward more precise, personalized, and sustainable development pathways. Despite these advances, critical research gaps remain, particularly in the limited mechanistic understanding of enzyme behavior in complex food matrices, the insufficient integration of multi-omics data with enzymatic process design, and the challenges associated with translating laboratory-scale enzymatic strategies into robust, data-driven, and scalable industrial applications.

## 1. Introduction

With the growing awareness of health and lifestyle transitions, consumer demand for foods that are nutritionally balanced, safe, convenient, and capable of delivering specific physiological benefits has increased rapidly. This shift has accelerated the development of functional foods and precision nutrition, while simultaneously posing new challenges to conventional food processing technologies. Enzyme technology, as an efficient, highly specific, and mild biocatalytic approach, operates under ambient temperature and pressure conditions and exhibits remarkable substrate specificity and regioselectivity. These characteristics enable substantial reductions in energy consumption, chemical residues, and undesired by-products, thereby providing a critical driving force for the realization of green, efficient, and sustainable food production systems [1,2]. Notably, such attributes are closely aligned with global policy initiatives, including the United Nations Sustainable Development Goals (SDGs), particularly those related to responsible consumption and production, climate action, and sustainable industrialization, as well as international strategies promoting green manufacturing and low-carbon food systems, further underscoring the strategic relevance of enzyme technologies in modern food processing.

In recent years, the application scope of enzyme technology in food processing and nutritional modulation has expanded markedly [3]. While numerous studies highlight the versatile roles of enzymatic catalysis in improving texture, flavor, and processing efficiency, the degree of benefit and mechanistic clarity reported in the literature remain inconsistent. For example, although enzymatic crosslinking systems have been shown to modify protein network structures and improve gelation and elasticity in both dairy and plant-based formulations [4,5], some results indicate that crosslinking can also reduce solubility and increase aggregation in certain plant proteins, particularly under high enzyme activity or non-optimized conditions, which may compromise textural uniformity in finished products. This discrepancy suggests that enzyme selection and process parameters critically determine functional outcomes, yet systematic comparisons across studies are still limited. Lipases and proteases are widely reported to facilitate the targeted generation of flavor precursor compounds, enriching sensory complexity in fermented foods [6]. However, the sensory effects are often highly substrate-dependent, and certain enzymatic treatments have been associated with the formation of off-flavors or excessive bitterness in protein hydrolysates, a problem that remains underexplored in comparative studies. Previous research demonstrates that extensive hydrolysis of lentil protein using Alcalase and Novozym significantly increases protein solubility under acidic conditions and alters particle size distribution and viscosity, thereby improving processing performance [7]. Contrarily, other studies have observed that extensive hydrolysis can weaken gel networks and reduce emulsification capacity, indicating that the purported processing benefits are not universally applicable across protein sources or hydrolysis intensities. Such contrasting findings underscore the need for more nuanced evaluations that relate enzyme specificity, hydrolysis degree, and functional outcomes. From a nutritional enhancement perspective, enzymatic reactions are widely credited with improving the absorption and bioavailability of proteins, minerals, and dietary fibers, as well as degrading antinutritional factors such as phytic acid and trypsin inhibitors [8,9,10]. While these improvements are promising, many studies rely predominantly on in vitro digestion models, and the translation of enhanced bioavailability to in vivo physiological effects remains inadequately validated. Moreover, the efficiency of enzyme-assisted extraction of polyphenols and antioxidant compounds from grape pomace has been shown to exceed that of conventional solvent methods [11]. It can vary significantly depending on enzyme type, substrate composition, and extraction conditions, highlighting a lack of standardized metrics for evaluating extraction performance. Proteolytic hydrolysis has been shown to modify protein structures in ways that enhance both functionality and nutritional value. For example, mushroom proteins treated with alkaline proteases reportedly show increased soluble protein fractions and higher release of small peptides and free amino acids during simulated gastrointestinal digestion, with corresponding gains in digestive efficiency [12]. However, not all protease treatments yield uniformly positive effects; some result in excessive peptide fragmentation, leading to reduced functionality in applications requiring structured networks. Furthermore, although enzymatic hydrolysis can disrupt protein–phytate complexes and other antinutritional structures, thereby potentially improving amino acid and mineral bioavailability [10], the extent of these benefits is often modest and highly context-dependent, emphasizing the need for comparative studies with control treatments and across diverse food matrices. At the level of functional innovation, enzyme-directed hydrolysis and glycosyl transfer reactions have enabled the efficient production of bioactive peptides, functional oligosaccharides, and rare sugars [13,14]. Nevertheless, challenges persist in scaling these processes industrially, including enzyme cost, product purification, and regulatory considerations, which are sparsely discussed in the current literature. While enzyme-assisted extraction has facilitated high-value utilization of natural bioactive compounds such as polyphenols and flavonoids, including enhanced anthocyanin recovery from eggplant peel with increased antioxidant activity compared to traditional methods [15,16], few studies address the stability and bioactivity retention of these extracts during downstream processing and storage. Moreover, enzymatic treatments of legume proteins have yielded hydrolysates with markedly distinct functional properties, including solubility, foaming capacity, and antioxidant activity, underscoring the high tunability of enzyme-based strategies in the rational design of functional foods [17]. However, observed variability across studies points to a broader lack of consensus on optimized reaction conditions, analytical methods, and performance benchmarks, which currently limits the direct comparability and practical adoption of enzyme technologies in functional food design.

Despite the well-recognized advantages of enzyme technology in texture modulation, flavor development, nutritional enhancement, and functional ingredient production, its translation from laboratory research to large-scale industrial application remains constrained by intertwined economic, technical, and engineering challenges. High development and production costs associated with enzyme discovery, protein engineering, and industrial fermentation particularly limit the cost-effectiveness of highly specific enzymes such as tailored proteases, glycosyltransferases, and regioselective lipases [18,19,20]. At the same time, many native enzymes exhibit insufficient stability under industrially relevant conditions, including elevated temperature, shear stress, pH fluctuations, and complex food matrices rich in lipids, polysaccharides, and inhibitory compounds, resulting in rapid activity loss and poor reproducibility. These limitations are further exacerbated at the process level, as industrial food systems are characterized by high viscosity, heterogeneous composition, multiphase structures, and competing substrates, which significantly reduce mass transfer efficiency and compromise the transferability of bench-scale optimization strategies to industrial environments, leading to pronounced batch-to-batch variability [21]. Moreover, enzymatic reactions are highly sensitive to subtle process fluctuations, and minor deviations in temperature, pH, or residence time can disproportionately affect hydrolysis degree, flavor precursor formation, and product distribution, complicating large-scale process control [22,23]. In addition, functional components generated through enzymatic routes often require intensive downstream separation and stabilization, while structural changes during storage, transportation, and digestion may further undermine functional consistency and economic feasibility [24,25]. Collectively, these constraints indicate that the industrial implementation of enzyme technology is governed less by intrinsic catalytic performance than by the ability to achieve a balanced integration of cost-efficiency, operational stability, scalability, and reproducibility within realistic and complex food matrices.

This review systematically summarizes key advances in the application of enzyme technology to process optimization, nutritional enhancement, and functional food development, with an emphasis on underlying mechanisms and practical evidence. Furthermore, future perspectives are discussed, highlighting the roles of protein engineering, metabolic engineering, and intelligent reaction control in advancing enzymatic catalysis. Improvements in enzyme molecular design, immobilization carrier optimization, and real-time process monitoring are expected to enhance catalytic stability and enable continuous processing, thereby laying a solid foundation for the broader adoption of enzyme technology in industrial food manufacturing. Ultimately, advances in enzyme technology are anticipated to support innovation and upgrading of the food industry by providing both theoretical insights and technological solutions for precision nutrition and functional food development.

## 2. Optimization of Food Processing Technologies: Improving Quality and Efficiency

### 2.1. Texture Modification and Protein Restructuring

The application of enzyme technology in food texture modification has expanded considerably in recent years, particularly in dairy products, meat products, soy-based foods, and plant-based meat analogs, where it plays a critical role in texture regulation. Texture attributes are closely associated with sensory perception and mouthfeel, and thus directly influence consumer acceptance, especially in plant-based foods, where replicating the fibrous structure, juiciness, and chewiness of conventional animal-derived products remains a key determinant of market success. Nevertheless, despite its substantial potential, achieving precise texture control across different food matrices remains technically challenging. In particular, the rational design of efficient enzyme combinations for diverse protein sources, as well as the mitigation of undesirable side effects arising during enzymatic treatments, continues to represent a major focus of current research.

Transglutaminase (TGase) is among the most extensively utilized enzymes in the food industry. It catalyzes covalent crosslinking between the γ-amide groups of glutamine residues and the ε-amino groups of lysine residues in proteins, resulting in the formation of interchain crosslinks that markedly enhance food texture [26]. The effectiveness of TGase in dairy, meat, and soy-based products has been well documented. In dairy systems, TGase has been shown to increase gel strength, water-holding capacity, and structural stability in products such as yogurt and cheese [27,28]. In meat products, TGase modulates protein conformation and promotes the exposure of reactive crosslinking sites, thereby enhancing protein binding capacity and elasticity and improving the sensory properties of sausages [29,30]. In soy-based foods, TGase catalyzes the formation of ε-(γ-glutamyl) lysine isopeptide bonds between glutamine and lysine residues, facilitating crosslinking between 7S and 11S protein subunits to form high-molecular-weight polymers, which significantly enhance the water-holding capacity and gel strength of tofu [31]. Collectively, these findings demonstrate that TGase is effective across dairy, meat, and plant-based systems. However, existing studies also highlight several key factors influencing final textural outcomes, including the degree of hydrolysis, the type of enzyme employed, and protein molecular weight distribution [32]. Excessive hydrolysis of soy protein may disrupt protein structure and consequently impair gelation performance. Therefore, maintaining structural integrity while precisely controlling the degree of hydrolysis is critical for achieving effective enzymatic crosslinking.

With the rapid rise of plant-based meat products, traditional processing technologies originally developed for animal-derived proteins are increasingly being adapted to plant-based systems. In this context, enzymatic crosslinking strategies have demonstrated distinct advantages. Laccase, in particular, has attracted growing attention for texture modulation in plant-based meat analogs. By catalyzing the crosslinking of aromatic residues and other macromolecular components in plant proteins, laccase alters intermolecular interactions and promotes the formation of more compact three-dimensional networks, thereby improving the texture and elasticity of plant-based meat products [33]. Specifically, laccase can oxidize phenolic compounds and sulfur-containing amino acid residues in proteins, leading to the formation of dityrosine bonds or the promotion of disulfide bond formation, which enhances texture, water-holding capacity, and mouthfeel [34]. Alizadeh et al. [35] reported that the addition of laccase during bread production catalyzed crosslinking between flour proteins and feruloylated arabinoxylans, generating a gluten-like three-dimensional network that improved elasticity and cohesiveness while reducing hardness. Similarly, peroxidases have been widely investigated for their ability to induce protein structural remodeling through the catalysis of covalent crosslinking between phenolic compounds and proteins, thereby increasing the hardness, elasticity, and water-holding capacity of tofu [36]. Compared with TGase, which primarily catalyzes amide bond formation between specific amino acid residues, laccase offers broader substrate versatility and is particularly suited to phenolic-rich plant matrices, although its activity is more sensitive to processing conditions and may result in more complex crosslinking methods. These findings collectively demonstrate the considerable potential of enzymatic crosslinking technologies in the texture improvement of plant-based foods. However, despite the pronounced effects of laccase and peroxidase on plant protein texture, further optimization is required in practical applications to balance enzyme dosage, reaction time, and temperature, and to minimize undesirable side reactions. In addition, in the production of plant-based fermented dairy alternatives, enzyme addition can disrupt overly dense protein gel networks, resulting in softer and smoother textures [37]. In fish processing, the inhibition of calpain activity has been shown to preserve protein structure and water-holding capacity, thereby delaying texture softening [38]. Through precise control of enzymatic treatment conditions, customized texture modification can be achieved to enhance both product functionality and consumer acceptance. For example, freeze–thaw enzyme infusion techniques can effectively soften vegetable tissues while maintaining their original appearance, offering promising approaches for the development of texture-modified foods tailored to aging populations [39,40].

Overall, the application of enzyme technology in food texture modification is moving toward increasingly precise and customized strategies (Table 1). By optimizing enzyme combinations and processing parameters, effective texture improvement can be achieved across diverse protein sources, thereby meeting the growing consumer demand for high-quality food products. From an industrial perspective, the scalability of such enzymatic approaches will depend on their robustness, cost-effectiveness, and compatibility with existing food processing operations. Future research should further explore synergistic effects among enzymatic crosslinking systems, the combined use of different enzyme classes, and the integration of enzymatic treatments with complementary technologies such as ultrasound and freezing, to achieve optimal outcomes in food texture modification.

**Table 1:** Utilization of enzyme technology for texture improvement in various food categories.

Enzyme	Treatment Conditions	Doses and Scale	Kind	Effects	Reference
Cellulase + amylase	55 °C; Cellulase pH 3.5; Amylase pH 5.5	Total enzyme concentration: 0.1% (*w*/*w*); Lab scale	Cassava	Effectively softened cassava.	[41]
Sesame protein isolate + TGase	-	TGase dosage: 0.6% (*w*/*w*); Lab scale	Gluten-free batter and cake	Increased batter viscosity and cake specific volume; reduced density and baking loss; improved texture and moisture retention; delayed staling (slow down aging).	[42]
Crude bromelain enzyme	40 °C; 35 min; pH 4.5–5.5	Pineapple protease extract: 3.0% (*v*/*v*); Lab scale	Cottage cheese	High protein, low fat, suitable texture and meltability, higher yield; could serve as a feasible alternative to rennet.	[43]
Cellulase	50 °C; 4.5 h; pH 5.0	Cellulase activity: 1.6 IU/mL; Lab scale	Brown rice	Reduced the cooking time and comprehensively improved its texture, flavor, and overall acceptability.	[44]
Cellulase, polygalacturonase and amylase	50 °C; 0.5, 1, 2, 3, 4 h	Enzyme dosage: 0.1% (*w*/*w*); Lab scale	Parsnip	Softened hardness and gave it a smooth texture.	[45]
Papain enzyme	4 °C; 0, 3, 6, 24 h	Papain dosage: 1.0% (*w*/*w*); Lab scale	Pork loin	Significantly improved tenderness.	[46]
β-glucosidase, xylanase	30 °C; 48 h	Lab scale	Wheat bran dough and bread	Degraded insoluble fiber and increased soluble components; released phenolics and ferulic acid, which enhanced nutritional value and antioxidant capacity; improved the dough’s gas-holding capacity, which increased bread volume and improved texture.	[47]
TGase	90 °C; 1 h	Optimal TGase dosage: 300 mg/g SPI; Lab scale	Plant-based meat	Exhibited a texture and thermal stability close to those of pork fat, significantly reduced brittleness, and decreased cooking loss.	[26]
Papain, bromelin, pepsin and pancreatin	55 °C; 1, 2, 3, 4, 5, 6, 7, 8 h	All enzymes: 0.075% (*w*/*v*); Lab scale	Horse meat	Tenderized horsemeat and significantly reduced its hardness and chewiness.	[48]
TGase, laccase	-	Lab scale	Plant-based meat	Softened the texture and maintained stability during cold-chain transport, long-term storage, and various cooking methods.	[49]

### 2.2. Flavor Modulation and Enhancement

**Figure 1:**
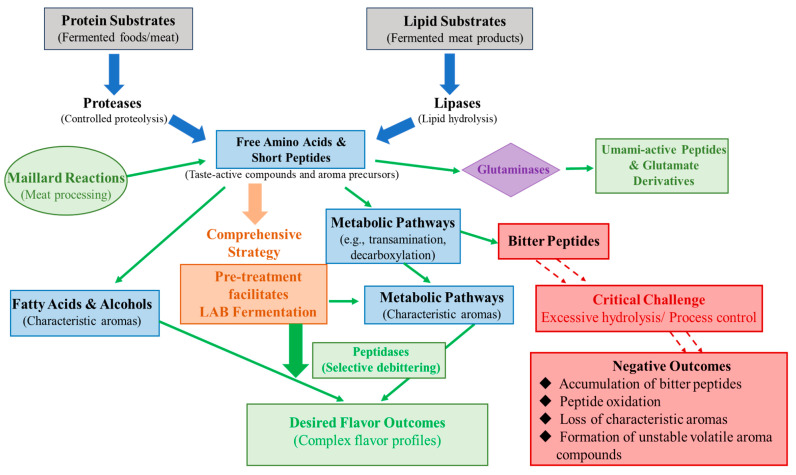
Enzyme-assisted pathways and control strategies for flavor development in fermented meat products.

Enzyme technology has emerged as a key approach for regulating the generation and transformation of flavor compounds, driving flavor research in foods from empirically driven processing toward precision-oriented design. However, major challenges remain, including how to exploit different classes of enzymes to achieve controlled release and conversion of key flavor precursors, while simultaneously enhancing desirable sensory attributes and suppressing excessive hydrolysis, side reactions, and adverse effects arising from processing conditions. Moreover, with the increasing demand for healthier products such as low-salt and low-fat foods, enzyme-based flavor compensation strategies have become an important emerging research focus. In fermented foods, proteases and lipases constitute the primary enzymatic systems responsible for flavor development [50,51]. Moderate proteolysis promotes the accumulation of free amino acids and short peptides, thereby accelerating cheese ripening and enhancing umami perception (Figure 1) [50]. Xiang et al. [52] proposed a combined strategy of “pre-enzymatic hydrolysis followed by lactic acid bacterial fermentation,” demonstrating that pretreatment with proteases or lipases facilitates lactic acid fermentation, significantly intensifies sourness and umami in cheese, and further converts amino acids through metabolic pathways such as transamination and deacidification, leading to more complex and enriched flavor profiles. Similarly, in meat processing, free amino acids and small peptides generated during enzymatic hydrolysis not only act as taste-active compounds themselves but also serve as key precursors for Maillard reactions [53]. For example, lipases in fermented meat products hydrolyze lipids to generate fatty acids and alcohols with characteristic aromas, effectively enhancing flavor complexity [54]. These advantages, however, depend critically on strict control of hydrolysis conditions. Excessive hydrolysis readily leads to the formation of bitter peptides, oxidative products, or undesirable volatile compounds, highlighting the narrow and sensitive window for enzymatic flavor regulation. At the molecular level of flavor control, peptidases and glutaminases exhibit higher specificity. Peptidases can selectively remove bitter peptides, thereby improving the flavor of plant proteins and fermented foods [55,56]. Glutaminase enhances umami intensity by promoting the formation of umami-active peptides and glutamate-related derivatives [57]. The effectiveness of these enzymes is highly dependent on substrate structure and precise cleavage site recognition, and their applicability across different protein systems requires further systematic evaluation.

Meanwhile, esterases have shown considerable promise in the green synthesis and reconstruction of key aroma compounds. During cheese ripening, esterases not only hydrolyze lipids to generate aroma precursors but also catalyze the synthesis of precursors into final flavor molecules [58]. Compared with conventional chemical synthesis of flavor compounds, esterase-catalyzed reactions are more environmentally friendly and can be conducted at lower temperatures, thereby avoiding undesirable reactions associated with high-temperature processing and improving flavor safety [59]. Nevertheless, a lack of consensus regarding substrate specificity, reaction kinetics, and controllable application currently limits the full industrial potential of esterases, as reflected by the pronounced variability in catalytic efficiency toward different ester substrates, difficulties in predicting flavor profiles under complex food matrices, and challenges in maintaining stable and reproducible enzyme performance during process scale-up. In the context of healthier food formulations, enzyme-assisted flavor compensation has emerged as a novel strategy. By modulating flavor components through enzymatic reactions, it is possible to improve sensory quality without increasing salt or fat content. For instance, flavor compounds generated via enzymatic hydrolysis can partially substitute for added salt, thereby reducing sodium levels without compromising taste [60]. Similarly, lipases can be used to modulate the flavor of reduced-salt foods by promoting lipid hydrolysis and oxidation, effectively compensating for flavor losses associated with salt reduction [61]. In addition, Tang et al. [62] demonstrated that combined enzyme treatments could modulate egg yolk protein structure and significantly reduce the formation of undesirable aldehydes, such as hexanal, heptanal, octanal, and benzaldehyde, thereby improving the flavor and texture of heat-induced egg yolk gels. In fermented foods, enzymes produced by Lactobacillus plantarum, including glycoside hydrolases and glycosyltransferases, can degrade substrates to generate flavor-related metabolites such as γ-aminobutyric acid and α-ketoglutarate, enhancing aroma and taste [63]. Enzymatic hydrolysis can also effectively release flavor compounds from edible mushrooms, allowing their use as natural flavor enhancers [60]. Despite these advantages, enzymatic reactions in plant- and fish-based systems may also introduce off-flavors, such as fishy or rancid notes, or excessively promote Maillard reactions, leading to negative sensory outcomes. These observations underscore the need for a more precise balance among substrate selection, reaction conditions, and downstream processing when applying enzymatic flavor compensation strategies [53,64]. Overall, enzyme technology is driving flavor modulation from a “process-driven” toward a “mechanism-driven” paradigm (Table 2). Future research should focus on elucidating synergistic mechanisms among different enzyme classes, constructing systematic models for the generation and transformation of flavor molecules, and exploring the integrated application of enzyme technology with fermentation, thermal processing, and emerging physical treatments to achieve greater controllability and predictability in flavor development.

**Table 2:** Applications of enzyme technology for flavor modulation and enhancement in food products.

Enzyme	Treatment Conditions	Doses and Scale	Kind	Effects	Reference
Cutinase, cellulase and pectinase	30 °C; 24 h; pH 6.5	Cutinase: 0.5 g/L, cellulase: 0.1 g/L, and pectinase: 0.01 g/L; Lab scale	Cherry tomatoes	The final product developed more intense and harmonious fruity, floral, and sweet aromas, and its flavor quality far surpassed that of traditional preserved fruits.	[65]
(Cellulase, hemicellulose and pectinase) + Protease	50 °C; 6 h; pH 5.0	Total enzyme activity: 8000 U; Lab scale	Sunflower oil	Increased the content of heterocyclic compounds, particularly O-heterocycles such as 2,3-dihydro-3,5-dihydroxy-6-methyl-4H-pyran-4-one (DDMP) and furfural derivatives.	[66]
Pectinase + α-amylase	-	Enzyme dosage: 1.0% (*w*/*w*); Lab scale	Zingiber officinale	Promoted the conversion of gingerol to shogaol and zingerone, enhancing the spicy and caramel flavors; it also facilitated the production of citronellal and citral; and significantly reduced the content of amino acids associated with bitterness.	[67]
Neutral protease + Flavourzyme	55 °C; 24 h	Neutral protease: 0.4% (*w*/*w*), flavor protease: 0.4% (*w*/*w*), and lipase: 0.1% (*w*/*w*); Lab scale	Cheese	Avoided the accumulation of bitter peptides and increased the content of flavor precursors (leucine, valine, isoleucine).	[68]
Straw mushroom endogenous enzyme	Room temperature—40 °C; pH 5.1–7.5	Lab scale	Cantonese sausage	Promoted lipid oxidation to generate small volatile molecules such as aldehydes, ketones, and alcohols, and significantly enhanced protein hydrolysis, leading to a substantial increase in total free amino acid content.	[69]
Protease	30–37 °C; 46 h	Pilot scale	Soy sauce	Degraded proteins, starch, and lipids, generating flavor precursors and final flavor compounds.	[70]
Protease, lipase	-	Lab scale	Fermented fish	Generated flavor precursors such as free amino acids (e.g., glutamic acid, alanine, leucine, and lysine).	[71]
Bromelain	50 °C; 3 h; pH 6.0	Enzyme activity: 9704 U/g, enzyme dosage: 10,323 U/g rice bran protein; Lab scale	Brown rice protein	Endowed it with a desirable flavor profile, including umami, rice-like aroma, and sweetness.	[72]
Papain + Neutral protease + Flavourzyme	50 °C; 3 h; pH 7.0	Lab scale	Lentinus edodes	Generated small molecular peptides and free amino acids, while simultaneously optimizing the amino acid composition and volatile compound profile, thereby endowing it with an optimal flavor.	[73]
α-amylase + Glucoamylase + Acid protease	-	α-Amylase: 6.3 U/g rice, glucoamylase: 94 U/g rice, acid protease: 7.7 U/g rice; Lab scale and pilot scale	Chinese rice wine	The content of free amino acids increased significantly, with a markedly greater rise in umami and sweet amino acids than in bitter ones. The levels of alcohols and esters were also substantially enhanced.	[74]

### 2.3. Improvement of Processing Efficiency and Yield

In modern food industries, improving processing efficiency and yield while preserving product quality and nutritional integrity is essential for advancing green manufacturing and sustainable development. Enzyme technology, characterized by high selectivity, mild reaction conditions, and environmental compatibility, is increasingly replacing traditional processing approaches that are energy-intensive and chemically aggressive. Nevertheless, the practical application of enzymatic processes remains challenged by system complexity, limited enzyme stability, and difficulties in process optimization. Achieving synergistic interactions within multi-enzyme systems and ensuring precise process controllability, while enhancing yield and maintaining the functional and nutritional properties of raw materials, has therefore become a central focus of current research. In fruit and vegetable processing, enzymatic depolymerization technologies have been shown to markedly improve juice yield and clarity. Enzymes such as cellulases, pectinases, and xylanases act synergistically to degrade cell wall components, reduce system viscosity, and facilitate juice release, while promoting the transfer of dietary fibers and polyphenolic compounds into the soluble phase, thereby enhancing both juice yield and nutritional value [75,76]. Through such enzymatic treatments, fruit resources can be utilized more efficiently, with reduced physical damage to pulp tissues compared with conventional mechanical pressing. Moreover, enzymatic hydrolysis enables the “migration” of nutrients from discarded pomace into juice fractions, contributing to the high-value utilization of agricultural products [77]. Compared with single-enzyme systems, multi-enzyme formulations provide complementary actions that significantly enhance reaction efficiency and minimize mechanical disruption of cellular structures. Variations among different substrates are primarily attributed to differences in cell wall complexity and non-starch polysaccharide content, which necessitate substrate-specific optimization of enzyme ratios and reaction times. Enzymatic processing also demonstrates distinct advantages in the valorization of plant proteins and animal by-products. Controlled or limited hydrolysis can improve the solubility and functional properties of oilseed meals and fish by-product proteins, thereby expanding their applicability in food systems. This approach relies on precise control of the degree of hydrolysis to prevent excessive degradation, while inducing structural loosening that enhances protein hydrophilicity and interfacial activity [78,79]. Differences in substrate recognition and cleavage specificity among enzyme sources result in hydrolysates with distinct nutritional profiles, flavor characteristics, and bioactivities. Overall, rational control of hydrolysis degree and reaction conditions is essential for achieving effective high-value conversion of by-products. In vegetable oil processing, enzymatic degumming technologies remove phospholipids, polysaccharides, and protein impurities through targeted hydrolysis, leading to efficient gum removal and improved oil quality. Compared with conventional chemical degumming, enzymatic approaches avoid acid or alkali residues and thermal damage, while improving oil purity and clarity and significantly reducing energy consumption and environmental impact [80].

The advantages of enzymatic methods are further reflected in their capacity to replace conventional organic solvent-based extraction processes, thereby enabling truly green processing routes. Traditional solvent extraction methods not only pose environmental risks but may also compromise product safety due to solvent residues. In contrast, enzyme-catalyzed reactions exploit the high selectivity and efficiency of enzymes to selectively degrade target components in plant matrices, reducing dependence on organic solvents such as petroleum ether and ethanol and consequently minimizing environmental pollution and health risks. In addition, the combined use of enzymes and ultrasound for starch modification has been shown to significantly enhance starch stability, transparency, and solubility, while minimizing the use of hazardous chemicals and reducing energy consumption. Similarly, integrating enzymatic hydrolysis with high-pressure processing can further improve the conversion efficiency of fish by-products into high-value protein hydrolysates, leading to substantial gains in processing yield and economic benefits [81]. With the advancement of digitalization and intelligent manufacturing technologies, real-time monitoring and smart control of key parameters, including temperature, pH, and enzyme activity, enable precise optimization of enzymatic processes, thereby improving processing efficiency while reducing energy waste. In summary, enzymatic processing is not only a means of increasing yield and efficiency but also provides precise control over raw material structures, thereby laying the foundation for subsequent release, transformation, and functionalization of nutritional components. Process optimization and nutritional enhancement are not independent objectives; rather, they are highly coupled within enzymatic systems, collectively supporting sustainable and value-added food production.

## 3. Enhancing Nutritional Value of Foods: Improving Bioavailability and Health Benefits

During food processing, improvements in processing efficiency and yield often directly influence the retention, structural state, and distribution of nutrients within food systems. Conventional high-intensity processing methods can increase yield but are frequently accompanied by nutrient losses or structural damage. In contrast, enzyme-assisted processing, through mild and controllable reaction conditions, enhances raw material utilization while creating favorable conditions for nutrient release, structural modification, and the generation of bioactive components. Therefore, building upon advances in processing efficiency and yield optimization, a further systematic examination of enzyme technology in enhancing food nutritional value, promoting nutrient absorption, and supporting health-related functions is of significant theoretical importance and practical relevance.

### 3.1. Improving the Bioavailability of Core Nutrients

**Figure 2:**
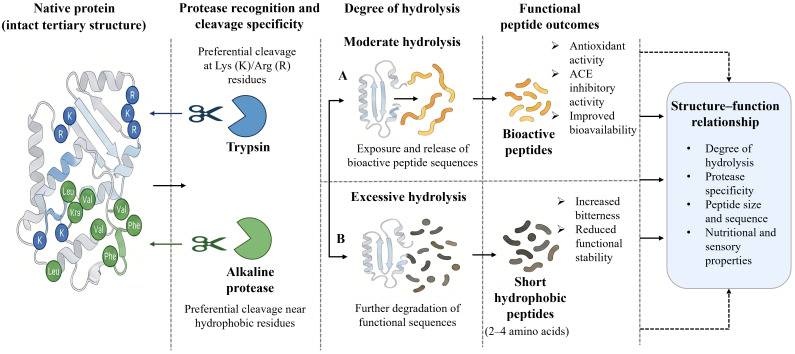
Protease–substrate interactions and cleavage-site specificity regulating the generation and functionality of bioactive peptides.

Within food systems, many essential nutrients, including proteins, polysaccharides, and minerals, are often present in complex macromolecular or bound forms, which limits their absorption and utilization in the human body. To enhance the bioavailability of these core nutrients, enzymatic degradation has been widely applied as an efficient, mild, and controllable biotechnological approach, particularly in infant formula, foods for the elderly, and sports nutrition products. Through enzymatic reactions, macromolecular nutrients such as proteins and polysaccharides can be converted into smaller molecular forms that are more readily digested and absorbed, thereby improving their bioavailability (Figure 2). However, differences and ongoing debates remain regarding the mechanisms of action of different enzymes, as well as the relationships between product structural characteristics and biological activity. Proteins, as major components involved in physiological structure and metabolic regulation, exhibit digestion and absorption efficiencies that are influenced by molecular conformation, amino acid sequence distribution, and processing conditions. Enzymatic hydrolysis alters protein tertiary and quaternary structures through specific enzyme–substrate recognition and cleavage at defined peptide bonds, generating peptide fractions with distinct molecular weight distributions. Numerous studies have demonstrated that moderate enzymatic hydrolysis not only enhances protein digestibility but also releases bioactive peptides with antioxidant, antimicrobial, or antihypertensive activities [82]. This process largely depends on the selective cleavage of peptide bonds adjacent to hydrophobic or charged amino acid residues, allowing functional sequences that are otherwise buried within the protein matrix to become exposed. In plant proteins, enzymatic hydrolysis improves nutritional quality by modifying molecular structures and converting high-molecular-weight proteins into low-molecular-weight bioactive peptides with enhanced antioxidant and antihypertensive properties [83,84]. For animal-derived proteins, such as milk and fish proteins, Liang et al. [85] reported that enzymatic hydrolysis not only disrupts allergenic epitopes and reduces the allergenicity of bovine milk, but also converts proteins into small peptides and free amino acids, thereby enhancing overall nutritional value. Similarly, enzymatic treatment of fish proteins converts macromolecular proteins into small peptides that exhibit improved digestibility and release bioactive peptides with antioxidant and antihypertensive activities [79]. These studies consistently indicate that the degree of hydrolysis, enzyme type (e.g., trypsin, alkaline proteases), and resulting molecular weight distribution collectively influence peptide bioactivity. Small peptides (<3 kDa) are more readily absorbed by cells and generally exhibit higher bioavailability [61]. However, excessive hydrolysis (DH > 10%) may generate peptides that are too short to effectively stabilize interfaces, leading to a decline in functional properties [52]. Moreover, enzyme systems such as alkaline proteases and trypsin display markedly different reaction characteristics depending on the substrate, underscoring the importance of substrate-specific enzyme selection and process optimization.

**Figure 3:**
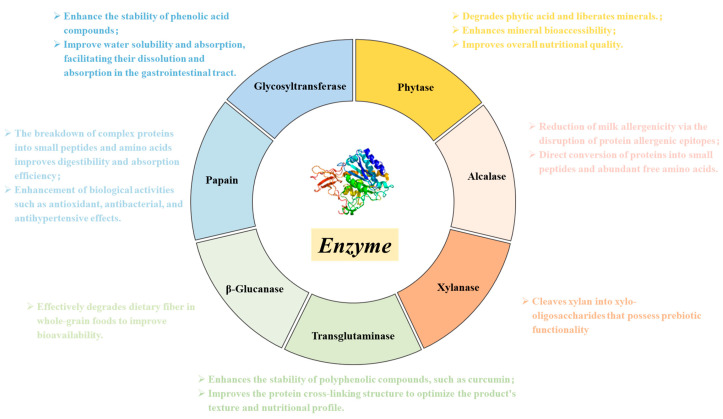
Enhancement of bioavailability of key nutrients in food through enzyme technology.

Beyond proteins, polysaccharide-based nutrients such as dietary fibers also exhibit limited bioavailability due to their complex macromolecular structures. Polysaccharide-degrading enzymes, including β-glucanase and xylanase, enhance bioavailability by degrading cell wall components and releasing soluble oligosaccharides, such as xylo-oligosaccharides and glucooligosaccharides (Figure 3). For example, β-glucanase can convert barley dietary fiber into sugars that are directly metabolized by yeast [86], whereas xylanase primarily acts on xylans by hydrolyzing β-1,4-xylosidic linkages in the main chain, generating xylo-oligosaccharides with prebiotic functionality and thereby improving bioavailability [87,88]. The application of these enzymes not only enhances dietary fiber utilization in whole-grain foods but also contributes to a reduced risk of certain chronic conditions, including constipation and intestinal disorders. Furthermore, the synergistic integration of physical assistance technologies with enzymatic treatments offers new opportunities to enhance nutrient bioavailability. For instance, ultrasound-assisted enzymatic hydrolysis can accelerate enzyme–substrate interactions, increase reaction rates, and improve yields of bioactive peptides, providing innovative strategies for functional food development [89]. Overall, future research should move beyond optimization of individual process parameters toward systematic design approaches. Strategies such as multi-enzyme synergy, enzyme immobilization, and coupling with physical fields should be employed to achieve precise control over nutrient structural modification and functional generation. In parallel, the integration of multi-omics approaches is essential for elucidating the intrinsic relationships between enzymatic hydrolysis products and human nutrient absorption, thereby establishing a theoretical foundation for precision nutrition and intelligent food processing.

However, structural modification and nutrient release alone are insufficient to fully enhance nutritional value and health-promoting properties. Antinutritional factors commonly present in food systems, as well as potentially harmful compounds formed during processing, may still limit nutrient absorption or pose health risks. Therefore, in addition to improving the bioavailability of core nutrients, targeted enzymatic degradation of antinutritional factors and harmful substances is necessary to achieve the synergistic enhancement of nutritional quality and food safety.

### 3.2. Degradation of Antinutritional Factors and Foodborne Harmful Compounds

**Figure 4:**
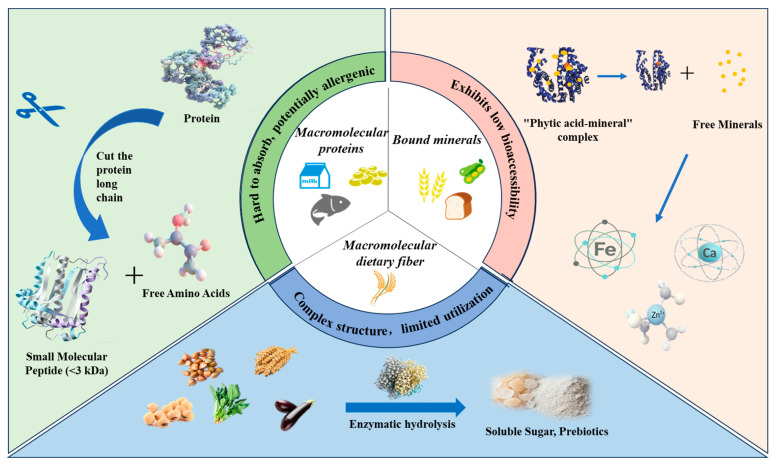
Mechanism and application of enzyme technology for targeted elimination of antinutritional factors and potential harmful substances in food.

Antinutritional factors and potential harmful compounds are critical determinants of food safety and nutrient bioavailability, particularly in plant-derived foods and thermally processed products. As illustrated in Figure 4, enzymatic degradation has emerged as a precise, mild, and efficient strategy for the targeted removal of antinutritional factors such as phytic acid, tannins, and trypsin inhibitors, as well as potential hazards including allergens. Nevertheless, how to enhance food safety and nutritional quality through selective enzymatic treatments remains a key challenge in practical applications. In recent years, accumulating evidence has demonstrated that the application of specific enzymes can effectively mitigate the adverse effects of these compounds, thereby ensuring both food safety and nutritional value. Phytic acid, a ubiquitous antinutritional factor in cereals, legumes, and nuts, readily chelates essential minerals such as iron, zinc, and calcium to form insoluble complexes, severely impairing mineral absorption. Phytase catalyzes the hydrolysis of phosphoester bonds in phytic acid, releasing bound minerals and thereby improving their bioavailability. The application of phytase has been shown to significantly reduce phytic acid levels and enhance mineral absorption without adversely affecting sensory attributes [90]. For instance, Poorvisha et al. [91] employed wheat phytase to dephytinize legume flour, resulting in a marked improvement in iron and zinc bioaccessibility. Similarly, Park et al. [92] reported that the addition of alkaline phytase to whole wheat bread significantly decreased phytic acid content while substantially increasing the solubility of key minerals, particularly iron and calcium, without altering the original processing conditions. However, phytase activity is highly dependent on pH, temperature, and substrate structure, leading to variable performance across different food matrices. Future research should therefore focus on the development of thermostable phytases with broad pH tolerance, as well as the application of immobilization or encapsulation technologies to enhance enzyme stability and industrial applicability. Tannins, a class of polyphenolic compounds widely distributed in fruits, tea, and other plant-based foods, are responsible for astringency and exhibit pronounced antinutritional effects. Enzymatic degradation studies have shown that ester bonds within tannins can be hydrolyzed by specific enzymes, thereby reducing the concentration of astringent compounds in juices and improving both sensory quality and nutritional value [93]. In addition, trypsin inhibitors are commonly present in legumes and nuts. Although they confer resistance to pests and pathogens in plants, they can interfere with protein digestion and amino acid utilization in humans. Targeted enzymatic treatments, such as vacuum impregnation combined with enzymatic hydrolysis, have been demonstrated to significantly reduce trypsin inhibitor activity, thereby improving the nutritional quality and digestibility of plant-based foods [64]. While these approaches highlight the potential of enzyme technology to enhance bioavailability and digestibility, precise control over enzyme dosage, reaction time, and degradation pathways remains an important area for further investigation.

Acrylamide, a potentially carcinogenic compound formed during high-temperature processing, is commonly detected in fried and baked foods such as potato chips and biscuits. Asparaginase has been shown to effectively convert asparagine into aspartic acid, thereby significantly reducing acrylamide formation during thermal processing [94]. In vegetable oil refining, lipases catalyze the rearrangement of fatty acids within triglycerides, which can effectively reduce the formation of trans fatty acids and consequently lower associated health risks [66]. Moreover, phospholipase A1 and monoacylglycerol lipase can completely hydrolyze phospholipids into water-soluble glycerophosphates, enabling efficient degumming and substantially reducing the levels of monoacylglycerols, which are key precursors of 3-monochloropropane-1,2-diol (3-MCPD) esters and glycidyl esters in vegetable oils, thereby enhancing oil safety [80]. Regulatory frameworks in major markets underscore the threat posed by acrylamide to public health. For example, the European Union has established benchmark levels for acrylamide in different food categories, including 750 μg/kg for potato products and 350 μg/kg for biscuits and crackers. A lower benchmark level of 150 μg/kg has been set for foods intended for infants and young children (https://eur-lex.europa.eu/eli/reg/2017/2158/oj/eng, accessed on 6 January 2026). In addition, the EU requires the implementation of mitigation measures to ensure that acrylamide levels are kept as low as reasonably achievable during production.

Food allergy represents an increasingly serious public health concern, particularly with respect to allergenic proteins in nuts and seafood, which pose significant risks to sensitized individuals. Enzymatic degradation has been widely adopted as an effective strategy to reduce allergenicity. Enzymatic hydrolysis cleaves peptide bonds and disrupts higher-order protein structures, fragmenting macromolecular proteins into smaller peptides and reducing or eliminating immunoreactive linear epitopes, ultimately lowering allergenic potential [95]. Furthermore, the combination of high-pressure processing with enzymatic hydrolysis has been shown to disrupt IgE-binding epitopes in nut proteins more rapidly and efficiently, achieving maximal allergenicity reduction [96]. High hydrostatic pressure-assisted enzymatic treatments have also demonstrated promising results in altering conformational epitopes of ginkgo seed proteins and shrimp tropomyosin [97]. With continued advances in enzyme engineering, enzymatic degradation technologies are expected to be applied to an expanding range of food systems, providing safer and healthier food products for consumers. In parallel, the integration of enzymatic treatments with emerging processing technologies, such as high-pressure processing and ultrasound, is anticipated to further improve efficiency and precision, thereby facilitating the transition of these approaches toward large-scale industrial implementation.

### 3.3. Structural Modification of Nutrients

With the growing emphasis on health-oriented nutrition, a central challenge in food processing is how to modulate the structure of nutrients through mild, efficient, and precise approaches so as to better align with human digestion and metabolism. Enzyme-mediated structural modification, owing to its high specificity and controllability, has been recognized as a key technological strategy for improving the nutritional quality of lipids and carbohydrates. In lipid modification, considerable attention has been devoted to mimicking the characteristic triacylglycerol structures of human milk fat to enhance the physiological suitability of infant formula. Numerous studies have demonstrated that lipase-catalyzed regiospecific interesterification can restructure triacylglycerol molecules to generate OPO-type lipids that more closely resemble those in human milk. When applied to infant formula, such structured lipids not only optimize fatty acid composition but also enhance immune function and promote the absorption of fat-soluble vitamins, thereby improving overall nutritional quality for infants [98,99]. Similar to lipids, enzymatic modification has shown pronounced effects in the structural remodeling of carbohydrates. Enzyme-assisted treatments can induce molecular reorganization of starch, and the coordinated action of amylases and debranching enzymes has been shown to markedly increase the contents of resistant starch and slowly digestible starch. This structural shift effectively lowers the glycemic index (GI) of starch, making it more suitable for populations with specific health needs, such as individuals with diabetes [100,101]. Although the extent of modification varies among starch sources, most studies converge on a common mechanism: enzymatic action alters chain-length distribution and branching architecture, rendering starch less susceptible to rapid hydrolysis by digestive enzymes. Moreover, enzymatic modification is frequently combined with physical pretreatments, such as mechanical disruption, to further expose reactive sites and enhance modification efficiency, highlighting multi-process coupling as an emerging trend for improving the precision of carbohydrate structural regulation.

At the same time, enzymatic glycosylation has achieved notable progress in enhancing the antioxidant activity and stability of polyphenolic compounds. Glycosylation catalyzed by glycosyltransferases can protect reactive functional groups of polyphenols, reduce oxidative degradation, and thereby significantly improve their stability. In addition, glycosylation enhances water solubility, facilitating intestinal absorption and ultimately increasing bioavailability [102]. Such modifications not only improve the nutritional value of polyphenols but also provide the food industry with more stable and health-promoting natural antioxidants. It is worth noting that both the glycosylation site and the type of sugar moiety exert a substantial influence on the resulting antioxidant activity, underscoring the need for systematic comparative studies in this area. Furthermore, several enzymatic modification strategies have focused on improving the nutritional accessibility of plant-derived proteins and non-starch polysaccharides. In oilseed meals such as palm kernel cake, high levels of non-starch polysaccharides act as antinutritional barriers that hinder nutrient digestion and absorption. Targeted application of xylanase and mannanase can selectively disrupt fiber structures, thereby improving nutritional quality [103]. Likewise, alkaline proteases can destabilize higher-order protein structures, facilitating the release of short peptides and enhancing functional properties [104]. A shared feature of these approaches is the targeted deconstruction of structural barriers to improve nutrient availability. However, given the complexity of substrate sources, the effectiveness of enzymatic modification varies widely among different fibers and proteins, indicating that deeper insights into the coupling between substrate structural characteristics and enzyme specificity are required. Overall, enzyme-mediated structural modification demonstrates substantial potential for optimizing a wide range of nutrients, including lipids, carbohydrates, polyphenols, and proteins. Its primary advantages lie in mild processing conditions, high reaction specificity, and the capacity for directed structural control. Although existing studies have revealed several common principles, the mechanisms underlying structure function variability remain to be further integrated and elucidated. Future progress in this field will depend on the coordinated advancement of enzyme engineering, process integration, and multiscale structural characterization, ultimately enabling precise nutrient modulation and facilitating the expanded application of functional foods in the context of precision nutrition.

## 4. Development of Functional Foods: Creating New Health Value

With the continuous rise in global consumer health awareness and the widespread recognition of the concept of “food as medicine,” functional foods are evolving from conventional nutritional supplements toward a central avenue of food innovation. Beyond meeting basic nutritional requirements, these products deliver additional health benefits through their bioactive constituents [105]. In advancing food functionality and precision nutrition, enzyme technology has been identified as a pivotal biomanufacturing tool [106]. Compared with traditional physicochemical processing methods, enzyme-catalyzed reactions operate under mild conditions, exhibit high substrate specificity, and are environmentally benign, thereby enabling more efficient liberation and utilization of the nutritional and health-promoting potential inherent in natural resources [107]. The following sections focus on the major application areas of enzyme technology in functional food development, including the targeted enzymatic production of bioactive peptides, the enzymatic synthesis of functional carbohydrates, and the enzymatic extraction and modification of natural bioactive compounds.

### 4.1. Targeted Production of Bioactive Peptides

**Figure 5:**
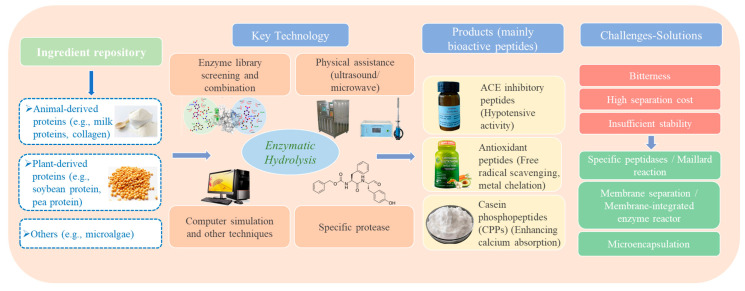
Targeted production and challenges solutions of bioactive peptides.

Bioactive peptides are low-molecular-weight peptide fragments composed of 2–20 amino acids linked by peptide bonds and are known to exert specific physiological functions [108,109]. These peptides are typically encrypted within the primary structures of animal, plant, or microbial proteins and remain biologically inactive in their intact protein forms. Only upon hydrolysis by specific proteases can they be activated and released, thereby exhibiting diverse health-promoting effects, including antihypertensive, antioxidant, anti-inflammatory, mineral absorption-enhancing, and immunomodulatory activities [108,110]. In the context of food and health applications, it is important to distinguish functional foods from nutraceuticals or dietary supplements: functional foods are consumed as part of the normal diet and deliver health benefits beyond basic nutrition within a conventional food matrix, whereas nutraceuticals are typically concentrated, purified bioactive preparations administered in non-food dosage forms such as capsules, tablets, or powders. Compared with chemical hydrolysis or microbial fermentation, enzymatic hydrolysis offers superior specificity and controllability, enabling the efficient generation of target bioactive peptides under mild reaction conditions [111]. The targeted enzymatic production of bioactive peptides encompasses several critical stages, ranging from the selection of suitable protein substrates at the upstream level, through the core enzymatic activation during controlled hydrolysis, to the downstream strategies required to address industrial-scale challenges. As illustrated in Figure 5, this technological route typically begins with animal- or plant-derived proteins. Through the precise selection and rational combination of protease systems, specific functional peptide sequences can be selectively released. Subsequently, downstream processing approaches, such as membrane separation and microencapsulation, are employed to overcome stability and scalability constraints, ultimately enabling the efficient incorporation of bioactive peptides into functional food products.

#### 4.1.1. Protein Substrate Sources and Enzyme Selection

Bioactive peptides can be derived from a wide range of protein sources, as nearly all proteins can serve as precursors. Early research predominantly focused on animal-derived proteins, particularly milk proteins and marine collagen, which are abundant and possess balanced amino acid profiles. These proteins are commonly used for the production of ACE-inhibitory peptides, antioxidant peptides, and immunomodulatory peptides [112,113,114,115]. Studies have shown that pepsin and trypsin can simulate gastrointestinal digestion, hydrolyzing αs1-casein to release casein phosphopeptides and related peptides, which activate GABAA receptors and exhibit sedative and anxiolytic effects [116]. Composite enzyme systems combining alkaline protease, flavourzyme, and papain are often employed for marine collagen hydrolysis, generating low-molecular-weight peptides that promote collagen synthesis and provide photoprotective effects [114,117]. With the rise of sustainable development and plant-based diets, plant-derived proteins have increasingly become the focus of bioactive peptide research. Soy protein, as a representative source, can be hydrolyzed by pepsin and trypsin to yield peptides that enhance intestinal cholesterol excretion and exhibit antioxidant activity [118,119]. More recently, emerging plant proteins such as pea, chickpea, rice, and hemp have demonstrated potential, with hydrolysates showing immunomodulatory, antioxidant, anti-diabetic, and neuroinflammatory inhibitory activities [120,121,122,123]. Additionally, microalgae proteins, such as those from spirulina and duckweed, exhibit notable antioxidant and anti-aging properties following enzymatic hydrolysis [124,125].

Enzyme selection has shifted from traditional empirical screening toward more precise strategies involving “enzyme library screening and combination optimization” to improve peptide production efficiency. High-throughput screening can evaluate the cleavage efficiency of various proteases on specific substrates, enabling optimization of hydrolysis conditions to obtain bioactive peptides with higher activity [126,127]. Pre-hydrolysis of whey protein using alkaline protease followed by flavourzyme treatment significantly increased both the yield and activity of ACE-inhibitory peptides, providing a promising strategy for future bioactive peptide development [128,129]. Moreover, the integration of artificial intelligence-based enzyme screening is emerging as a powerful trend to further accelerate the identification of optimal enzymes and cleavage sites.

#### 4.1.2. Enzymatic Production and Functional Validation of Key Bioactive Peptides

Targeted enzymatic hydrolysis is central to the production of bioactive peptides, involving the selection of appropriate enzymes and substrates as well as the optimization of reaction conditions to maximize the yield, purity, and bioactivity of the desired peptide sequences. ACE-inhibitory peptides function by suppressing the activity of angiotensin-converting enzyme (ACE), thereby blocking the conversion of angiotensin I to angiotensin II and ultimately lowering blood pressure [130]. The mechanism relies on peptide sequences, particularly those containing proline, lysine, and aromatic amino acids, binding to the active site of ACE and competitively inhibiting its catalytic function, resulting in vasodilation and reduced sodium retention [131]. Enzyme selection favors those capable of specifically cleaving peptide bonds at the C- or N-terminal of these residues. The introduction of computational simulations and sequence-prediction tools, such as UniProtKB and BIOPEP databases, has provided novel strategies for peptide design. These approaches allow for the screening of potential bioactive sequences and prediction of the optimal enzymes required for their release, thereby shortening the development cycle. For instance, in corn and chickpea protein sequences, simulated hydrolysis using papain and alcalase demonstrated more efficient release of di- and tri-peptides containing terminal aromatic or hydrophobic residues, which enhance hydrogen bonding and hydrophobic interactions with ACE, leading to improved inhibitory activity [132,133].

Antioxidant peptides exert their effects by scavenging free radicals, chelating metal ions, or inhibiting oxidase activity. Sequences rich in histidine, tyrosine, and tryptophan, as well as hydrophobic or aromatic residues, can donate electrons or hydrogen atoms to stabilize free radicals or chelate transition metals, preventing Fenton reaction-induced hydroxyl radical formation [134,135]. Alkaline and neutral proteases are commonly employed to release these sequences. Studies have shown that combining enzymatic hydrolysis with physical-assisted techniques such as ultrasound or microwave treatment enhances hydrolysis efficiency. This synergistic effect arises from physical disruption of protein secondary and tertiary structures, exposing more hydrophobic sites, increasing enzyme–substrate contact, and accelerating mass transfer, thereby improving the degree of hydrolysis and antioxidant activity [136]. For example, ultrasound–microwave-assisted hydrolysis of beet and sweet potato proteins using Alcalase and Flavourzyme resulted in higher hydrolysis degrees and enhanced Fe^2+^ chelation, ·OH radical scavenging, and ORAC values compared with conventional enzymatic treatment [137]. In pumpkin seed protein, ultrasound-assisted enzymatic hydrolysis increased the degree of hydrolysis by 57.7%, while DPPH and ABTS antioxidant activities rose by 52.4% and 42.6%, respectively [138]. In milk proteins, microwave pretreatment increased hydrolysis efficiency, generated low-molecular-weight peptides, and enhanced antioxidant capacity [139].

Calcium-absorptive peptides are primarily derived from casein phosphopeptides within casein, containing clusters of phosphorylated serine residues capable of forming stable complexes with calcium ions. These complexes prevent calcium precipitation under neutral and alkaline conditions, thereby improving intestinal absorption [140,141]. The production of such peptides relies heavily on the specific cleavage by trypsin [140]. It can be observed that the selection of enzymes is closely related to the structural characteristics of peptides, as shown in Table 3. With the widespread application of cellular and animal models and advances in proteomics and peptidomics technologies, precise screening of active sequences and functional validation has become increasingly efficient. This progress facilitates the discovery of novel bioactive peptides with anti-inflammatory, antimicrobial, antidiabetic, neuroprotective, and cognitive-enhancing functions, providing new opportunities for functional peptide research.

#### 4.1.3. Industrialization Challenges and Emerging Solutions

Although enzymatic production of bioactive peptides holds considerable theoretical and practical potential, industrial-scale application faces multiple challenges, including pronounced bitterness of the products, high costs of separation and purification, and insufficient stability within food matrices and gastrointestinal environments [24,34]. Bitterness primarily arises from the accumulation of short-chain hydrophobic peptides. Traditional activated carbon adsorption can remove these bitter peptides but often results in concomitant loss of bioactivity [13]. The use of specific exopeptidases enables selective removal of hydrophobic amino acid residues at peptide termini, improving flavor while preserving functional activity [142]. In wheat gluten hydrolysates, the release of bitter peptides is highly correlated with the content of hydrophobic residues such as Leu, Ile, Val, Phe, and Tyr. Exopeptidases, including aminopeptidases and carboxypeptidases, can cleave these terminal hydrophobic residues, thereby reducing bitterness [143]. Leucine aminopeptidase LapA from Aspergillus oryzae preferentially removes N-terminal hydrophobic residues, effectively mitigating bitterness in milk casein hydrolysates [144].

Maillard reactions and enzymatic glycation have been explored to further improve flavor. These modifications covalently link reducing sugars to peptide amino groups, altering the spatial configuration of hydrophobic residues, masking bitterness receptor-binding sites, and simultaneously enhancing umami and taste stability. For instance, fish skin collagen hydrolysates reacted with xylose, ribose, glucose, and glucosamine via Maillard reactions exhibited markedly reduced bitterness and enhanced umami and salty tastes. The covalent conjugation between reducing sugars and peptides alters the exposure of hydrophobic residues responsible for bitterness, thus improving flavor [145]. In catfish muscle hydrolysates, glycation increased the proportion of peptides below 1 kDa and decreased those above 5 kDa, thereby lowering the content of bitter amino acid residues [146].

Membrane separation technology remains the most widely applied industrial method for peptide fractionation, exploiting size exclusion via semipermeable membranes to separate small peptides from larger substrates [147]. Emerging “membrane-integrated enzymatic reactors” couple hydrolysis and separation within a single system, where enzymes are immobilized on the membrane surface or within the reaction chamber. As the substrate flows through, continuous hydrolysis occurs; small peptides permeate through the membrane while larger substrates and enzymes are retained, enabling enzyme recycling and continuous operation, significantly enhancing production efficiency [148]. The stability of bioactive peptides within food matrices and gastrointestinal conditions is another critical concern. Microencapsulation has emerged as an effective strategy to protect peptides [149,150]. Using wall materials such as whey protein, alginate, and chitosan, protective coatings can be formed to shield peptides from pH variations, heat, and enzymatic degradation, allowing targeted release and enhanced bioactivity [151,152].

Beyond technical and production bottlenecks, regulatory approval represents a significant hurdle for the commercialization of bioactive peptides. Currently, the definition, classification, and safety assessment standards for peptide ingredients remain inconsistent across different jurisdictions, leading to complex and lengthy approval processes. Due to the structural complexity of bioactive peptides, ensuring batch-to-batch consistency is challenging, which poses difficulties for quality control and ingredient identification. Furthermore, regulatory agencies often demand more rigorous human clinical evidence to substantiate the physiological efficacy and toxicological safety of peptides after gastrointestinal digestion. This requirement for high-level empirical evidence significantly increases the R&D costs and market entry barriers for functional foods and pharmaceutical ingredients.

### 4.2. Enzymatic Synthesis of Functional Carbohydrates

**Figure 6:**
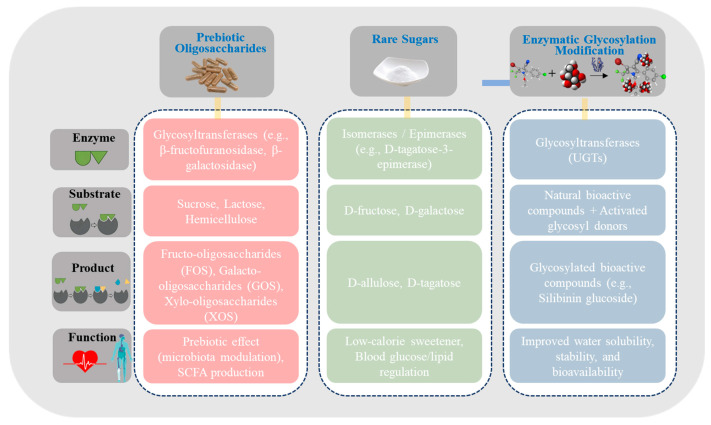
Enzymatic synthesis of functional carbohydrates.

Functional carbohydrates are bioactive sugars that exert significant regulatory effects on human health. Unlike conventional sugars, which primarily serve as energy sources, functional carbohydrates often possess low caloric content and exhibit prebiotic activity, glycemic regulation, and anti-cariogenic properties [153]. Traditional chemical synthesis of functional carbohydrates typically requires harsh reaction conditions and generates complex by-products, which is unfavorable for green manufacturing. In contrast, enzymatic synthesis, utilizing biocatalysts such as glycosyltransferases and isomerases, enables the efficient and selective production of structurally defined and functionally active carbohydrates from inexpensive and readily available sugar substrates under mild conditions [14,154]. The application of enzymatic methods in functional carbohydrate synthesis primarily focuses on three areas: the large-scale production of prebiotic oligosaccharides, biotransformation of rare sugars, and glycosylation modification of natural bioactive compounds. As illustrated in Figure 6, these three categories of functional carbohydrate synthesis differ in their core enzyme systems, substrate types, and the key functional attributes of the final products, collectively forming a comprehensive enzymatic platform for functional carbohydrate production.

#### 4.2.1. Prebiotic Oligosaccharides

The role of prebiotics in modulating gut microbial balance has been well established, particularly functional oligosaccharides such as fructo-oligosaccharides (FOSs) and galacto-oligosaccharides (GOSs). These prebiotics are selectively utilized by beneficial gut microbes, promoting their proliferation and consequently conferring positive effects on host health.

The enzymatic synthesis of FOSs primarily involves β-fructofuranosidase-catalyzed transfructosylation, transferring fructosyl units from sucrose to another sucrose molecule, generating a series of oligosaccharides with GFn structures. To enhance yield, enzymes with high transfructosylation-to-hydrolysis (T/H) ratios have been screened from extremophiles such as thermophilic and acidophilic microorganisms, and immobilization strategies have been employed to enable continuous reactions and reduce production costs [155,156]. GOSs, structurally similar to human milk oligosaccharides, play a pivotal role in infant gut microbiota development and are therefore widely incorporated into premium infant formula [157]. Enzymatic GOS synthesis relies on β-galactosidase-mediated transgalactosylation, in which the enzyme simultaneously hydrolyzes lactose and transfers galactosyl units to another lactose or galactose molecule, forming oligosaccharides with diverse glycosidic linkages [158]. Successful enzymatic production of prebiotic oligosaccharides requires careful coordination of the enzyme system and substrate conditions. Catalytically, β-fructofuranosidase active sites typically contain acidic residues (Glu, Asp) that act as nucleophiles or proton donors, forming enzyme–sugar complexes and intermediate oxocarbenium ions to enable fructosyl transfer [159]. Site-directed mutagenesis altering the hydrophobic/hydrophilic environment, modifying metal-ion cofactors, or reshaping substrate channels can optimize the T/H ratio and improve oligosaccharide yields [160]. From a process perspective, substrate concentration, enzyme carrier state (immobilized or free), and diffusion limitations interact dynamically. High substrate concentrations may drive transglycosylation but can also saturate enzyme active sites, favor hydrolysis, or create mass transfer limitations [161,162]. Optimizing immobilized carriers with tailored pore structures, hydrophilic–hydrophobic balance, and enzyme distribution enhances enzyme–substrate interactions and prolongs enzyme lifetime [163]. Additionally, multi-enzyme cascades that establish substrate-pull or intermediate-channeling pathways can overcome equilibrium constraints, achieving higher conversion rates [163].

The physiological effects of oligosaccharides depend not only on dosage but also on structural characteristics and gut metabolic pathways. Variations in glycosidic linkages (β-1,3, β-1,4, β-1,6) and chain lengths determine the selective utilization by gut microbes such as Bifidobacterium and Lactobacillus. For instance, GOS mixtures predominantly containing β-1,3 and β-1,6 linkages showed higher fermentation activity by Bifidobacterium, indicating linkage type influences prebiotic utilization efficiency [164]. In the colon, oligosaccharides are fermented by gut microbiota to produce short-chain fatty acids (SCFAs, e.g., acetate, propionate, butyrate), which activate host AMPK or Nrf2 signaling pathways, thereby regulating lipid metabolism, enhancing mucosal barrier function, and reducing inflammation [165,166].

Beyond FOSs and GOSs, newer prebiotics have been developed. Xylo-oligosaccharides (XOSs), derived from hemicellulose in agricultural by-products via xylanase hydrolysis, exhibit effective doses far lower than conventional prebiotics [167]. XOS fermentation in the colon produces SCFAs that selectively promote Bifidobacterium growth, lower intestinal pH, and enhance SCFA production, improving gut health and earning the description “super prebiotic” [168]. Short-chain oligosaccharides are rapidly absorbed in the upper gut, whereas longer chains delay fermentation, maintaining prebiotic effects in the distal colon [169,170]. Other prebiotics, including isomalt-oligosaccharides, chitosan oligosaccharides, and soybean oligosaccharides, can similarly be produced via enzymatic pathways [171,172].

#### 4.2.2. Rare Sugars

Rare sugars, naturally occurring in extremely low abundance, are characterized by low caloric content, mild sweetness, and minimal impact on blood glucose, making them ideal sucrose alternatives for obesity and diabetes management [173,174]. Enzymatic production typically employs a network of isomerases and epimerases to enable efficient reversible conversion between monosaccharides [175]. D-allulose, a representative rare sugar, has approximately 70% of the sweetness of sucrose but negligible caloric contribution, and can reduce postprandial blood glucose and improve lipid metabolism [176]. Its synthesis relies on D-tagatose-3-epimerase and D-allulose-3-epimerase-catalyzed isomerization of D-fructose [177,178], and D-allulose has been approved as a food additive in multiple countries for beverages, baked goods, and confectioneries [179].

D-Tagatose, another naturally occurring rare sugar with sucrose-like taste, provides only one-third of the caloric content of sucrose and exhibits prebiotic effects [180]. Its production starts from lactose hydrolysis by β-galactosidase to yield D-galactose, followed by L-arabinose isomerase-catalyzed conversion to D-tagatose [181].

Rare sugar synthesis generally involves isomerase-catalyzed transformations, which are reversible and often limited by equilibrium. Strategies such as cascade catalysis, product removal, or continuous substrate feeding can improve conversion efficiency [182]. Combining immobilized isomerases with microreactor technology increases enzyme–substrate contact, minimizes diffusion losses, and allows precise control of temperature and substrate gradients, enabling continuous high-efficiency operation.

The nutritional benefits of rare sugars derive from their structural uniqueness and metabolic pathways. Their stereochemistry reduces affinity for intestinal transporters and digestive enzymes, leading to slower absorption, decreased postprandial glucose fluctuations, and lower insulin responses [183]. Some rare sugars activate hepatic AMPK, promoting fatty acid oxidation while suppressing lipid synthesis, contributing to metabolic syndrome mitigation [184]. Additionally, rare sugars modulate gut microbiota composition, increasing SCFA production and conferring anti-inflammatory and intestinal barrier-protective effects [185].

The production and application of rare sugars not only reflect advances in biotechnology but also underscore their potential significance in health management. They are expected to play increasingly important roles in both the food industry and nutrition-based therapeutic strategies in the future.

#### 4.2.3. Enzymatic Glycosylation

Enzymatic glycosylation involves the transfer of activated sugar moieties to acceptor molecules under the catalysis of glycosyltransferases or certain glycosidases, providing an effective strategy to improve the physicochemical properties and bioactivity of natural compounds [186]. Many naturally occurring bioactive substances suffer from poor water solubility and low stability, limiting their applications in food systems. By introducing sugar moieties through enzymatic glycosylation, the solubility, stability, and bioavailability of these compounds can be markedly enhanced. For instance, the maize-derived glycosyltransferase UGT706F8 has been employed to glycosylate silibinin, yielding a high-yield, stable 7-O-β-d-glucoside form [187]. The introduction of sugar increases molecular hydrophilicity, facilitating dissolution and dispersion of previously poorly water-soluble compounds in aqueous food matrices [188]. Glycosylated bioactives exhibit improved nutritional functionality by enhancing stability, absorption, and enabling targeted release. The added sugar moieties increase molecular polarity and water solubility, enhancing stability in the gastrointestinal environment and reducing degradation or aggregation during digestion, thereby preserving bioactivity [189]. Additionally, glycosylation can improve interactions with intestinal transport proteins, promoting active absorption [190]. In some cases, glycosidic bonds can be cleaved by probiotics, gradually releasing the parent bioactive compound and prolonging its in vivo activity [191].

Glycosylation also contributes to enzyme efficiency by facilitating nucleotide sugar donor regeneration. Incorporating donor-regenerating enzymatic systems maintains constant sugar donor concentrations, significantly improving reaction yields [192]. Protein engineering strategies that modify the hydrophobicity of the glycosyltransferase substrate channel can enhance binding specificity toward particular polyphenol or flavonoid acceptors. Moreover, immobilization of glycosyltransferases supports continuous reactions and integrated product separation.

With advances in synthetic biology and metabolic engineering, complete glycosylation pathways can be constructed in engineered microbial strains. These pathways integrate all steps from activated sugar synthesis, glycosyl transfer, to acceptor molecule glycosylation within the microbial host, enabling efficient production of target glycosylated compounds [193].

### 4.3. Enzymatic Extraction and Bioactivity Enhancement of Natural Bioactive Compounds

**Figure 7:**
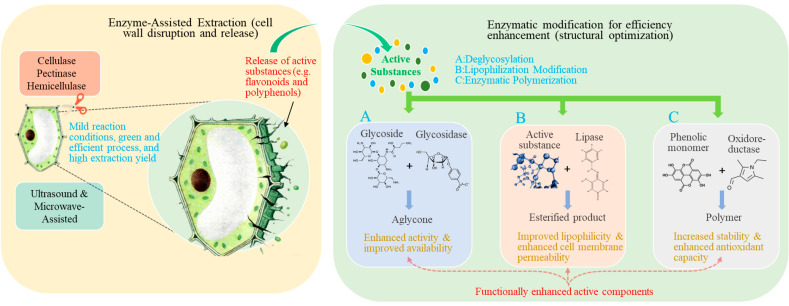
Enzymatic extraction and bioactivity enhancement of natural bioactive compounds.

Plants are rich in health-promoting functional compounds, including polyphenols, flavonoids, anthocyanins, and terpenoids. However, conventional extraction methods often rely on high temperatures or large volumes of organic solvents, which impose significant limitations. These thermolabile and solvent-sensitive compounds are prone to degradation during extraction and may also pose environmental pollution or residue concerns. Enzyme-assisted extraction employs cell wall-degrading enzymes such as cellulases, hemicellulases, pectinases, and proteases to synergistically disrupt plant cell wall structures under relatively mild conditions, thereby facilitating the release of intracellular bioactive compounds. This approach not only improves extraction efficiency but also enhances product quality, offering a green, safe, and efficient alternative. The application of enzymatic technology in natural bioactive compound development is evolving from simple extraction assistance toward structural modifications that strengthen structure–activity relationships, as illustrated in Figure 7.

#### 4.3.1. Mechanisms and Advantages of Enzyme-Assisted Extraction

Plant cell walls are composed of a complex network of polysaccharides and proteins, including cellulose, hemicellulose, pectin, and structural proteins, which serve as the primary physical barrier impeding the release of bioactive compounds. Enzyme-assisted extraction (EAE) exploits the substrate-specific hydrolytic activity of enzymes to disrupt this network. Cellulases cleave cellulose microfibrils, pectinases degrade the pectic substances in the middle lamella, and hemicellulases break down hemicellulose, gradually loosening or even completely dismantling the cell wall. This significantly reduces the mass transfer resistance during compound diffusion into the solvent and avoids the thermal or pressure-induced degradation of heat-sensitive molecules. For instance, the combined application of pectinase, cellulase, and tannase in green pistachio shells increased the extraction yields of apigenin and luteolin by approximately 239% and 248%, respectively, under optimized enzyme conditions [194]. Similarly, in red grape pomace, the use of cellulase, hemicellulase, and pectinase enhanced total phenolic content (TPC) and antioxidant activity compared with conventional solid–liquid extraction [11].

EAE offers multiple advantages: it operates under mild conditions, preserving the structure and function of thermolabile compounds [11]; it typically uses water as the solvent, avoiding organic solvents and aligning with clean-label and green chemistry principles [195]; and enzyme specificity allows targeted release of compounds by selecting different enzyme types or combinations to act on specific regions of the cell wall [11]. Compared with high-temperature or organic solvent extraction, EAE achieves higher extraction efficiency within shorter times and enhances the bioavailability of active compounds, thereby improving the nutritional value fundamentally.

#### 4.3.2. Synergistic Application of Multi-Enzyme Systems

Given the structural complexity of plant cell walls, a single enzyme often cannot achieve complete disruption. Multi-enzyme systems are therefore critical. Rational combinations of cellulase, pectinase, xylanase (or hemicellulase), with optimized ratios and reaction conditions, can achieve synergistic effects and substantially improve the release of bioactive compounds. Studies have shown that extraction yields using enzyme cocktails can increase by over 50% compared with single-enzyme systems under the same conditions [196]. The benefit of multi-enzyme systems lies not merely in the mixture of enzymes but in their cooperative action. For example, pectinase first acts on the gel matrix of the middle lamella, loosening intercellular connections, which subsequently allows cellulase or xylanase to access microfibrillar regions more effectively, accelerating the overall cell wall degradation [197,198].

Customized enzyme cocktails tailored to the specific cellulose, pectin, and hemicellulose composition of the plant material can maximize extraction efficiency by precise control of enzyme type, ratio, reaction time, and temperature. For instance, α-L-rhamnosidase and β-d-glucosidase are commonly used for extracting and converting flavonoid glycosides in citrus peels, cleaving rhamnose residues to facilitate compound release and reduce bitterness [199]. Meanwhile, integrating enzyme-assisted extraction with physical intensification techniques has become a current trend to further enhance extraction efficiency. Ultrasound-assisted enzymatic extraction utilizes cavitation effects to improve enzyme–substrate contact and facilitate the diffusion of bioactive compounds, thereby achieving higher yields in shorter timeframes [200]. A study on ultrasound–enzyme combined extraction of phenolic compounds from grape pomace demonstrated that this method significantly outperformed conventional enzymatic extraction alone [201]. Similarly, microwave and high-pressure-assisted approaches can enhance cell wall disruption and accelerate bioactive compound release [202,203].

Multi-enzyme systems not only increase extraction yields but may also influence the release and modification status of active molecules. During cell wall breakdown, low-accessibility phenolics bound to the wall can be liberated. The mild enzymatic conditions minimize thermal or oxidative degradation, preserving more of the native active compounds, thereby enhancing both the quantity and bioactive quality for optimal nutritional functionality.

#### 4.3.3. Enzymatic Modification for Bioactivity Enhancement

The application of enzymes has gradually extended from compound extraction to structural modification and functional enhancement. Many plant bioactives exist as glycosides, whereas their corresponding aglycones often exhibit higher bioavailability and physiological activity [204]. Glycosidase-catalyzed deglycosylation can convert glycosides into more active aglycones. For example, ginsenoside Rb1 from Panax ginseng, after β-glucosidase hydrolysis, produces rare ginsenosides F2 and CK with significantly higher antitumor activity than the parent compound [205]. Similarly, enzymatic hydrolysis of soy isoflavone glycosides to aglycones enhances absorption and estrogenic activity [206].

Enzymatic modifications also include lipophilization, where lipases and proteases catalyze esterification of bioactives with fatty acids under low water activity or non-aqueous conditions. This introduces hydrophobic tails, enhancing lipid solubility, membrane permeability, and overall bioavailability [207]. For instance, catechin esters such as catechin octanoate and dodecanoate synthesized via lipase-mediated reactions exhibit superior antioxidant activity in lipid systems compared to the parent catechin [208].

Enzymatic polymerization has also been explored. Oxidoreductases (e.g., laccase, peroxidase) catalyze coupling or polymerization of phenolic compounds to generate higher-molecular-weight polymers with enhanced antioxidant capacity, thermal stability, and film-forming properties compared with monomers [209]. Reports indicate that lignin-derived phenolics esterified via lipase show improved hydrophobicity and antioxidant potential [210].

Overall, enzymatic modification, with its catalytic precision, mild operational conditions, and environmental friendliness, is shaping strategies for enhancing bioactive compounds in functional foods. From targeted protein hydrolysis and carbohydrate structural modification to reinforcement of plant secondary metabolites, enzyme catalysis not only increases product value but also provides key technological support for precision nutrition and personalized food development.

## 5. Challenges and Future Perspectives

### 5.1. Contributions and Value of Enzyme Technology

**Figure 8:**
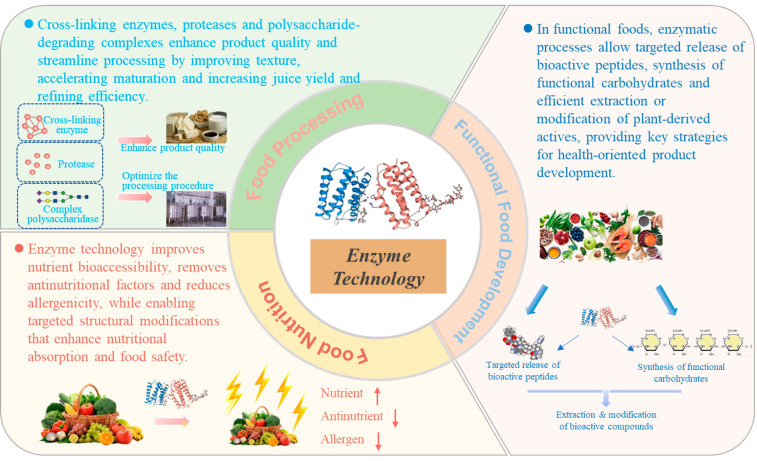
Applications of enzyme technology in food processing, nutritional enhancement, and functional food development.

As a precise, environmentally friendly, and highly efficient biocatalytic approach, enzyme technology is increasingly driving innovation in the field of modern food nutrition. Its applications span food processing, nutritional enhancement, and the development of functional foods, making it a key technological strategy for achieving both quality optimization and improvements in nutritional value (Figure 8).

In the food processing sector, enzyme technology serves as a key tool for improving textural properties and enhancing processing efficiency. Cross-linking enzymes, represented by TGase, laccase, and peroxidase, can catalyze specific protein cross-linking reactions, thereby markedly improving the gelation behavior, water-holding capacity, elasticity, and thermal stability of dairy products, meat products, soy-based foods, and plant-based meat analogs [211,212,213]. In terms of flavor development, the application of proteases and lipases accelerates the ripening of cheeses and fermented meat products, while the targeted use of peptidases and glutaminases effectively removes bitter peptides or generates umami-active peptides, significantly enhancing sensory quality and consumer acceptance [214,215]. With respect to processing efficiency, the synergistic application of multi-enzyme systems such as cellulases and pectinases increases juice yield and clarity, whereas in oil refining, enzymatic degumming processes, owing to their mild operating conditions, energy efficiency, and environmental friendliness, are progressively replacing conventional chemical methods [216].

From a nutritional perspective, the core value of enzyme technology lies in enhancing nutrient bioaccessibility and enabling the precise removal of undesirable factors. Proteases can gently hydrolyze macromolecular proteins into readily absorbable peptides and amino acids, making them particularly important in infant formula and elderly nutrition products. Phytases degrade phytic acid in cereals, releasing chelated micronutrients such as iron and zinc [217], while β-glucanases and xylanases convert dietary fibers into oligosaccharides with prebiotic activity [218]. In addition, enzyme-based approaches provide efficient strategies for controlling antinutritional factors and potential hazards by selectively degrading compounds such as phytic acid, tannins, and trypsin inhibitors, thereby minimizing interference with nutrient absorption [219,220,221]. By disrupting key epitopes of allergenic proteins, enzymatic treatments can effectively reduce food allergenicity [222]. Moreover, during high-temperature processing, the application of specific enzyme systems helps suppress the formation of acrylamide and trans fatty acids, contributing to improved food safety [223]. In terms of enzymatic structural modification, lipases have been employed to synthesize human milk fat analogs (OPO lipids), amylases to modulate starch digestibility for the production of slowly digestible starch, and glycosylation reactions to enhance the stability and solubility of polyphenolic compounds [224,225,226].

In the development of functional foods, enzyme technology represents an important pathway for implementing the concept of food as medicine and expanding health-promoting functions. Enzymatic processes enable the targeted production of bioactive peptides; by selecting specific proteases and controlling hydrolysis conditions, peptides with antihypertensive, antioxidant, and anti-inflammatory activities can be efficiently released from animal and plant proteins [227]. Enzyme-catalyzed glycosyl transfer reactions provide an efficient and sustainable route for the synthesis of functional oligosaccharides and low-calorie rare sugars [189]. EAE, using cell wall-degrading enzymes, allows the release of polyphenols, flavonoids, and other bioactive compounds from plant tissues under mild conditions [228]. Subsequent enzymatic deglycosylation or lipophilization can further enhance their bioavailability and functional performance [229].

Overall, owing to their mild reaction conditions, high substrate specificity, excellent catalytic efficiency, and sustainability advantages, enzyme technologies are progressively replacing conventional physicochemical methods. They are driving the food industry toward greater precision, greener production, and higher value addition, thereby providing strong support for the integrated advancement of food science and human nutrition and health.

### 5.2. Limitations and Challenges

**Figure 9:**
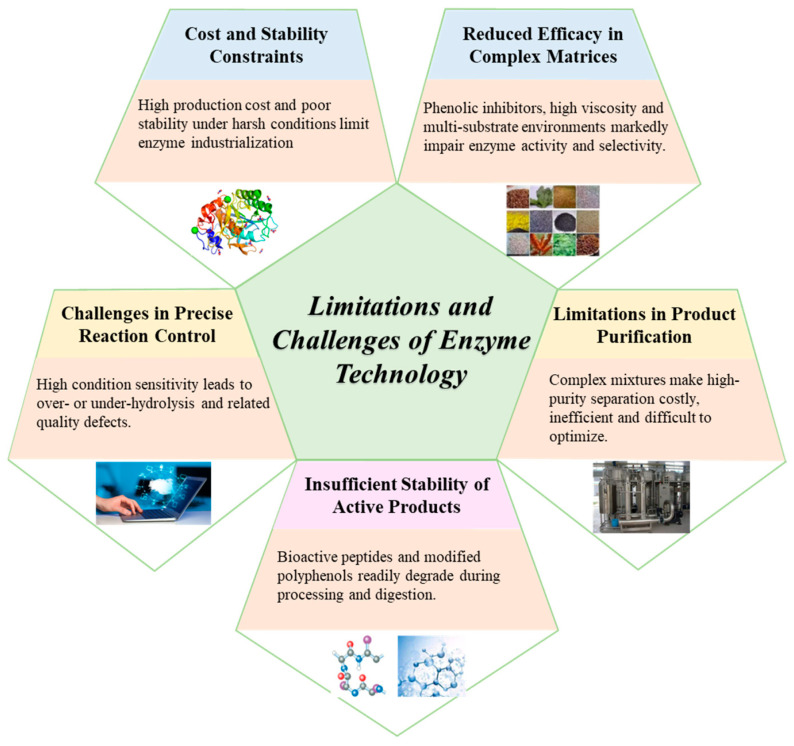
Limitations and challenges in the application of enzyme technology.

Despite the substantial progress achieved in both fundamental research and practical applications, the translation of enzyme technology from laboratory-scale studies to large-scale industrial production under complex and variable processing conditions still faces a range of technical and economic constraints, as illustrated in Figure 9.

Enzyme Cost and Stability. The high cost and limited stability of enzyme preparations remain major constraints on their industrial application. A techno-economic analysis of β-glucosidase production reported that, at an annual output of 88,000 kg, the unit production cost was approximately USD 316/kg; through simulated optimization of fermentation, induction strategies, and downstream simplification, the theoretical minimum cost could be reduced to around USD 37/kg [230]. In practice, however, compliance with food safety, purity, activity, and regulatory requirements often necessitates complex purification and stabilization steps, which substantially increase production costs. Moreover, most naturally derived enzymes retain structural integrity and catalytic activity only under mild pH and temperature conditions, making them poorly suited to the harsh processing environments commonly encountered in the food industry, such as high temperature, extreme pH, high salinity, or high pressure [231,232]. Although screening or molecular engineering can yield enzymes tolerant to extreme conditions, their development cycles are long, costs are high, and applicability is often limited. At present, neither production capacity nor market price is sufficient to support widespread large-scale implementation.

Reduced Performance in Complex Food Matrices. Food systems are considerably more complex than laboratory model systems. Naturally occurring phenolic compounds, tannins, and other inhibitory substances may bind to enzymes or interfere with their active sites, thereby reducing catalytic efficiency [220]. In addition, high-viscosity matrices, such as doughs or concentrated fruit pulps, impose severe mass-transfer limitations that hinder enzyme diffusion to substrate surfaces, resulting in reaction rates far lower than those observed in dilute solutions [233]. When multiple substrates coexist, competitive or non-specific reactions may further compromise selectivity and precision. It has been reported that enzymes exhibiting both high activity and efficiency under ideal dilute conditions often show markedly diminished performance in complex food matrices, leading to a substantial gap between laboratory results and industrial outcomes [234].

Difficulty in Precise Process Control. Enzymatic reactions, particularly hydrolytic processes, are highly sensitive to reaction time, substrate concentration, and enzyme dosage. Minor deviations can result in over- or under-processing, ultimately affecting product quality, including protein functionality, gelation behavior, emulsification, and foaming properties. In functional protein modification, moderate hydrolysis can improve gelation, whereas excessive hydrolysis disrupts secondary and tertiary structures, preventing effective gel network formation and reducing functionality [235]. For example, soy protein isolates subjected to mild enzymatic hydrolysis (degree of hydrolysis, DH ≈ 2.17%) produced hydrolysates with superior gel properties, characterized by more homogeneous networks, smaller pore sizes, and improved viscoelastic behavior and water-holding capacity. In contrast, higher DH values (3.12–6.61%) led to deteriorated gelation, increased turbidity, and reduced solubility [236]. Although hydrolysis can release flavor-active peptides, it may also generate large amounts of short-chain hydrophobic peptides that impart pronounced bitterness and reduce sensory acceptability [143]. Furthermore, these hydrolysis products may participate excessively in subsequent thermal processing via Maillard reactions, consuming key flavor precursors and ultimately weakening overall aroma intensity [237].

Challenges in Product Separation and Purification. For the industrial development of high-value functional foods such as bioactive peptides and enzyme-modified polyphenols, enzymatic reactions typically yield highly complex mixtures. Target bioactive compounds often require multiple downstream separation and purification steps, including chromatography, membrane filtration, or adsorption, to achieve adequate purity and activity. Although membrane-based and adsorption techniques have been improved, they often suffer from limited throughput and insufficient selectivity. Even activated carbon adsorption used to remove bitter peptides may concomitantly remove hydrophobic bioactive peptides, resulting in a loss of biological activity [238]. In applications demanding high purity and strong functionality, such purification processes are costly and inefficient, posing a major barrier to large-scale production.

Stability of Bioactive Products in Food Matrices. Even after successful production and purification, maintaining the stability and functionality of bioactive compounds during food processing, storage, and digestion remains challenging. Bioactive peptides and enzyme-modified polyphenols are susceptible to degradation or inactivation under heat treatment, pH fluctuations, or gastrointestinal digestion, limiting their ability to reach target sites and exert the intended physiological effects. Although microencapsulation and nano-delivery systems have been proposed to enhance stability and targeted delivery, these approaches often increase process complexity and cost. Achieving an optimal balance among stability, bioactivity retention, and economic feasibility remains difficult [150].

Overall, the broader application of enzyme technology in the food sector requires advances in enzyme molecular engineering, reaction system optimization, process scale-up, downstream separation, and delivery system design. Only by maintaining an appropriate balance between catalytic efficiency and economic viability can enzyme technology truly achieve sustainable and large-scale implementation in the food industry.

### 5.3. Future Perspectives

**Figure 10:**
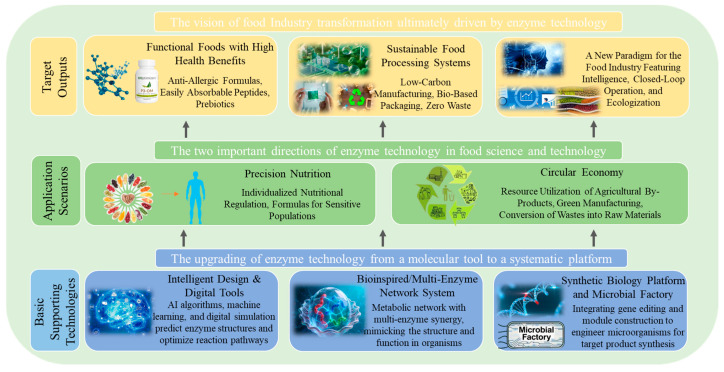
Future development trajectory of food enzyme technology.

To fully realize the potential of enzyme technology in the field of food nutrition, future developments will move beyond the screening of individual enzymes or localized optimization of process parameters, toward holistic innovation driven by deep multidisciplinary integration and system-level design. From intelligent enzyme design and innovation of reaction systems to precision-oriented applications and smart manufacturing, technological convergence and systematic development will define the future trajectory of food enzyme technology. As depicted in Figure 10, the future development of enzyme technology is systematically outlined, with particular emphasis on emerging strategies aimed at enhancing precision, robustness, and scalability. Progress in protein engineering, enzyme immobilization, and synthetic biology is expected to improve catalytic stability and specificity, while the adoption of intelligent process control and real-time monitoring will enable dynamic optimization under industrial operating conditions. Moreover, the integration of multi-omics datasets with enzymatic process design opens new avenues for elucidating structure, function and nutrition relationships, thereby supporting the advancement of precision nutrition and personalized food development.

#### 5.3.1. Future Enzymes: From Natural Discovery to Intelligent Design

Overcoming the intrinsic performance limitations of natural enzymes represents a critical starting point for innovation in enzyme-based technologies. The sourcing of enzymes is shifting from passive natural discovery toward proactive and intelligent design. Traditionally, industrial enzymes have been derived mainly from culturable microorganisms, which constitute only a small fraction of the total microbial diversity in nature. The emergence of metagenomics has fundamentally changed this paradigm by enabling the direct extraction of total DNA from environmental samples, such as soils, deep-sea sediments, and extreme ecosystems, followed by the construction of gene libraries. This approach allows access to the vast genetic reservoir harbored by the approximately 99% of microorganisms that are not readily culturable [239]. Metagenomic enzyme discovery strategies are generally categorized into function-driven and sequence-driven approaches. Function-driven screening focuses on enzymatic activity and directly identifies clones with target functions through high-throughput assays. For example, B. Oliva and colleagues employed this strategy to identify a series of putative cold-adapted enzymes from ikaite column sediments in Greenland, which show promising potential for low-temperature food processing applications [240]. In contrast, sequence-driven screening relies on high-throughput sequencing and bioinformatic analyses to identify candidate enzymes by detecting conserved domains within known enzyme families [241]. Using this approach, Wang et al. [242] identified a multifunctional cellulase (C5-Cel4) from sediments of the Aibi Lake salt deposit in Xinjiang, exhibiting both halotolerance and thermostability. Notably, this enzyme retained full activity under high-salinity and high-ionic-liquid conditions, highlighting its potential for biomass pretreatment applications.

However, enzyme discovery alone is insufficient, as industrial implementation often requires substantial structural and functional optimization. Artificial intelligence-driven rational design is bringing transformative changes to enzyme engineering. Conventional structural elucidation methods rely on time-consuming and costly crystallographic techniques, whereas AI-based models such as DeepMind’s AlphaFold2 and the RoseTTAFold framework developed by David Baker’s group can predict three-dimensional protein structures with high accuracy directly from amino acid sequences. These advances provide a robust structural foundation for large-scale rational enzyme modification [243]. When integrated with machine learning algorithms, large datasets linking sequence and function can be analyzed to construct nonlinear predictive models, enabling the estimation of how specific mutations may influence properties such as thermostability or catalytic efficiency [244]. More advanced generative AI models can even design entirely novel enzymes de novo, creating functional proteins that do not exist in nature and enabling on-demand, tailor-made biocatalysts [245].

At the same time, directed evolution remains a cornerstone strategy for improving enzyme performance. This approach involves generating large mutant libraries through random mutagenesis, followed by high-throughput screening to identify variants with enhanced properties. For instance, error-prone PCR-based modification of a Pseudomonas fluorescens lipase has been shown to significantly improve its thermal stability [246]. In recent years, droplet-based microfluidic technologies have increased screening throughput to the scale of millions or even tens of millions of variants, greatly enhancing the efficiency of directed evolution. Looking ahead, the integration of AI-guided prediction with directed evolution is expected to become a dominant paradigm, wherein AI informs precise mutation strategies and intelligent library design, while experimental iteration drives performance optimization. This synergistic approach is poised to substantially shorten development cycles and accelerate the emergence of next-generation, high-performance food enzymes.

#### 5.3.2. Production Modes: From Batch Processing to Continuous Manufacturing

Once high-performance enzyme molecules are obtained, their efficient utilization becomes a critical consideration. Conventional aqueous batch reactions suffer from inherent limitations, including difficulties in enzyme recovery, susceptibility to deactivation, and limited precision in process control, all of which constrain economic viability and operational efficiency. Advancing catalytic systems toward greater intelligence, integration, and sustainability represents a key trend in the industrialization of enzyme technology.

Enzyme immobilization constitutes a fundamental strategy for enabling enzyme reuse and enhancing operational stability. By anchoring enzymes onto insoluble supports through physical adsorption, covalent bonding, or entrapment, immobilization not only facilitates enzyme recovery but also improves tolerance to variations in temperature, pH, and solvent environments [18]. In recent years, emerging carrier materials such as magnetic nanoparticles, metal–organic frameworks (MOFs), and covalent organic frameworks (COFs) have attracted considerable attention due to their high specific surface areas, well-defined pore structures, and tunable physicochemical properties. Magnetic nanoparticles allow rapid separation under an external magnetic field, substantially reducing downstream separation costs [247]. MOFs and COFs can encapsulate enzyme molecules in a molecular cage like architecture, preventing enzyme leaching and deactivation while providing low-resistance diffusion pathways for substrates and products [248]. Moreover, the introduction of affinity tags onto enzymes via genetic engineering enables site-specific immobilization, ensuring optimal enzyme orientation on the support and thereby significantly enhancing catalytic efficiency and operational lifetime [249].

Building upon immobilization strategies, the development of multi-enzyme cascade systems represents an advanced paradigm in biocatalysis. These systems mimic natural metabolic pathways by spatially and functionally integrating multiple enzymes within a single reaction environment, enabling continuous conversion from substrates to final products without the need for intermediate isolation. This approach markedly shortens reaction routes and improves atom economy [250]. The effectiveness of such systems critically depends on the compatibility of reaction conditions among different enzymes and the optimization of their spatial organization. Studies have demonstrated that precise nanoscale positioning of multiple enzymes on DNA nanoscaffolds can significantly enhance intermediate transfer rates, giving rise to substrate channeling effects [251]. In the synthesis of functional carbohydrates, multi-enzyme systems have achieved efficient conversion of lactose to D-tagatose and enabled the construction of complex biosynthetic networks involving up to five coordinated enzymes [252].

In parallel, continuous-flow reaction technologies are increasingly replacing conventional stirred-tank reactors. Continuous-flow reactors operate by maintaining constant substrate input and product output, allowing substrates to react continuously within columns packed with immobilized enzymes [253]. Compared with batch processes, continuous-flow systems offer superior mass and heat transfer, precise control over residence time, and higher space–time yields [254]. Experimental studies have shown that immobilized enzymes can operate stably for tens of hours in continuous packed-bed reactors, with markedly improved conversion efficiencies [255]. When integrated with online monitoring and automated control systems, continuous-flow technology holds strong promise as a scalable production platform for high-value food ingredients, such as functional carbohydrates and bioactive peptides. Although challenges remain, including long-term enzyme stability and hydrodynamic optimization during reactor scale-up, the potential of continuous processing to enhance production efficiency and enable greener manufacturing is substantial.

#### 5.3.3. Enzyme Applications: From Universal Improvement to Precision Customization

Driven by innovations in enzyme sourcing and process optimization, the application of enzyme technology is evolving from general quality improvement toward precision nutrition and circular economy objectives.

In the context of precision nutrition, enzymes serve as molecular tools due to their high substrate specificity, enabling individualized dietary interventions based on genotype, metabolic profile, and gut microbiota composition. For infant nutrition, region-selective lipases can catalyze interesterification to synthesize OPO-structured fats that mimic human milk [256]. For individuals with diabetes, starch-modifying enzymes can produce slowly digestible or resistant starches, facilitating the development of low-glycemic staple foods [257]. For elderly populations, specific proteases can generate extensively hydrolyzed, easily absorbable bioactive peptides that promote muscle synthesis [258]. Enzyme technology can also enhance nutrient bioavailability by breaking down indigestible domains in plant proteins or degrading phytic acid in cereals [217,259]. Moreover, enzyme-sensitive delivery systems can be engineered to release bioactive compounds at targeted sites within the gastrointestinal tract [260]. Future applications may enable the modular synthesis of personalized prebiotics tailored to individual gut microbiota profiles, achieving precision nutritional interventions.

From a circular economy perspective, enzyme technology offers a green and efficient route for valorizing agricultural and food industry by-products. Materials such as rice husks, wheat bran, soybean residues, and fruit peels can be converted into high-value ingredients using enzymatic processes. For example, xylanases can convert wheat bran into XOSs [261], and ferulic acid in cereal processing by-products can be recovered enzymatically [262]. Protease hydrolysis of oilseed meals can generate functional proteins and bioactive peptides with antioxidant and immunomodulatory activities [263]. Cellulases and pectinases can disrupt plant cell walls to efficiently extract pectins, polyphenols, and natural pigments from fruit and vegetable residues [11,264]. Even traditional waste streams such as cheese whey can be transformed into whey protein powders, lactoferrin, and GOSs through combined enzymatic and membrane separation technologies [265]. Fish processing by-products can likewise be hydrolyzed enzymatically to produce high-value fish protein hydrolysates [266]. Beyond food applications, lipases can be employed to treat food wastewater with high organic loads at ambient temperatures, reducing energy consumption and secondary pollution [267]. Laccases have also been reported to catalyze the crosslinking of polyphenols with chitosan, forming high-performance biodegradable biofilms as alternatives to petrochemical plastics [268,269].

#### 5.3.4. Integration of Intelligent Technologies and Biological Systems

Future food enzyme engineering will constitute a highly integrated, multidisciplinary system science, merging digitalization, intelligence, and biomimicry. Artificial intelligence and automated design will underpin the entire enzyme engineering cycle. With tools such as AlphaFold2 expanding structural databases, machine learning models can more accurately predict enzyme catalytic behavior in complex food matrices. Landwehr et al. [270] employed machine learning to analyze tens of thousands of reaction datasets, optimizing the activity of an amide-synthesizing enzyme and achieving a 42-fold increase in catalytic efficiency, demonstrating the potential of digital models in enzyme optimization.

Simultaneously, the development of biomimetic enzyme systems will advance enzyme technology to higher levels. By spatially organizing multiple enzymes via protein scaffolds, carrier linkages, or vesicle encapsulation, synthetic metabolic networks can be established to improve reaction kinetics and substrate flux while minimizing diffusion losses of intermediates [271]. Studies indicate that, in the processing of complex agricultural by-products, the synergistic action of hydrolytic enzymes, oxidases, and microbial enzymes can be optimally configured within biomimetic systems to achieve efficient conversion [272].

Synthetic biology is expected to become central to enzyme innovation. By embedding AI-designed metabolic pathways into engineered microorganisms such as recombinant yeast or E. coli, high-value functional products can be efficiently biosynthesized [273,274,275]. Utilizing low-cost agricultural by-products as feedstocks, these approaches enable sustainable production of rare sugars, bioactive peptides, and modified natural compounds, transforming the food industry from conventional processing to intelligent biomanufacturing. This represents not only a higher stage in enzyme technology development but also a step toward a more precise and environmentally friendly food sector.

#### 5.3.5. Summary

Enzyme technology has emerged as a core enabling tool for improving food processing efficiency, enhancing nutritional value, and driving functional food innovation. Despite substantial progress, its large-scale industrial application remains constrained by several key bottlenecks, including limited enzyme stability under complex food matrices, high production and purification costs, and insufficient predictability of enzymatic behavior during scale-up. These challenges highlight that further development of enzyme technology requires not only optimization at the process level but also deeper integration with emerging interdisciplinary methodologies. In light of the limitations identified in the preceding sections, such as enzyme instability under industrial conditions, limited process controllability in complex food matrices, and challenges in translating laboratory-scale optimization to industrial implementation, future advances in enzyme technology for food nutrition should increasingly rely on integrated and data-driven strategies rather than isolated single-factor optimization.

AI is expected to play pivotal roles in shaping the next generation of enzymatic food processing. Advances in protein engineering, such as rational design and directed evolution, enable the development of enzymes with enhanced stability, broader operational windows, and tailored substrate specificity. Meanwhile, AI- and machine learning-based tools provide powerful platforms for enzyme screening, structure–function prediction, reaction condition optimization, and multi-enzyme system design. By leveraging large datasets generated from enzymatic reactions and food processing systems, AI-driven strategies can transform enzyme application from experience-based optimization to data-driven and predictive control.

Furthermore, the integration of multi-omics technologies and intelligent manufacturing systems will be critical for advancing enzyme technology toward precision nutrition and sustainable food production. Multi-omics approaches, including proteomics, metabolomics, and microbiomics, offer systematic insights into how enzymatic processing alters food structure, releases bioactive components, and influences digestion, absorption, and metabolic responses. At the industrial level, intelligent manufacturing concepts such as real-time sensing, process analytical technology, and digital twins enable dynamic monitoring and adaptive regulation of enzymatic reactions, ensuring consistent product quality while reducing energy consumption and resource waste. Collectively, the convergence of enzyme engineering, AI, multi-omics research, and smart manufacturing is expected to reposition enzyme technology from an auxiliary processing tool to a central driver of innovation in functional foods and precision nutrition.

## 6. Conclusions

With their high efficiency, specificity, and environmental compatibility, enzymes are becoming a key driver of innovation in the food industry and nutrition science. By improving texture and flavor during processing, enhancing bioavailability of core nutrients while degrading anti-nutritional factors, and enabling the production of bioactive peptides, functional sugars, and natural active compounds, enzymes permeate all stages of food production. Current research highlights that composite enzyme systems, immobilized enzymes, and intelligent control of enzymatic reactions offer new opportunities for industrial application. However, high cost, limited stability, and complexity of reaction systems remain key barriers to widespread adoption. Future efforts should integrate protein engineering, metabolic engineering, and process optimization to develop highly active, stable enzyme systems while enabling dynamic control of reactions and efficient product separation. Enzyme technology is poised to play an increasingly significant role in precision nutrition, functional foods, and sustainable processing, driving the food industry toward efficiency, safety, and ecological sustainability.

## Figures and Tables

**Table 3:** Functional properties of bioactive peptides generated by enzymatic hydrolysis.

Peptide Type	Key Enzymes and Techniques	Key Amino Acid Features	Main Function and Mechanism
ACE-inhibitory peptides	Enzyme: • Papain • Alcalase (particularly for corn and chickpea proteins)	• Proline • Lysine • Aromatic AAs (particularly hydrophobic or aromatic residues located at the terminal positions)	Antihypertensive activity: By inhibiting ACE, the conversion of angiotensin I to angiotensin II is suppressed, thereby promoting vasodilation and decreasing sodium retention
Antioxidant peptides	Enzyme: • Alcalase • Neutral proteases • Flavourzyme Assistive Processing Technology: • Ultrasound • Microwave (Physical-assisted treatments can disrupt the native protein structure, leading to the exposure of buried hydrophobic sites)	• Histidine • Tyrosine • Tryptophan • hydrophobic or aromatic residues	Free radical scavenging/metal chelation: Free radicals are stabilized through electron or hydrogen atom donation, while transition metal ions are chelated to prevent Fenton reactions, thereby inhibiting oxidative enzyme activity.
Calcium-absorptive peptides	Enzyme: • Trypsin (The production of such peptides primarily relies on the specific cleavage activity of trypsin.)	• Clusters of phosphorylated serine (casein phosphopeptides originating from casein proteins)	Promotion of calcium absorption: By forming stable complexes with calcium ions, calcium precipitation under neutral or alkaline conditions is prevented, thereby enhancing intestinal calcium absorption.

## Data Availability

No new data were created or analyzed in this study. Data sharing is not applicable to this article.
